# Transcriptional memories mediate the plasticity of cold stress responses to enable morphological acclimation in *Brachypodium distachyon*

**DOI:** 10.1111/nph.16945

**Published:** 2020-10-26

**Authors:** Boris F. Mayer, Jean‐Benoit Charron

**Affiliations:** ^1^ Department of Plant Science McGill University 21, 111 Lakeshore Sainte‐Anne‐de‐Bellevue Canada

**Keywords:** *Brachypodium distachyon*, chromatin, cold acclimation, growth, phenotypic plasticity, stress, transcriptional memory

## Abstract

- Plants that successfully acclimate to stress can resume growth under stressful conditions. The grass *Brachypodium distachyon* can grow a cold‐adaptive morphology during cold acclimation. Studies on transcriptional memory (TM) have revealed that plants can be primed for stress by adjusting their transcriptional responses, but the function of TM in stress acclimation is not well understood. We investigated the function of TM during cold acclimation in *B. distachyon*.
- Quantitative polymerase chain reaction (qPCR), RNA‐seq and chromatin immunoprecipitation qPCR analyses were performed on plants exposed to repeated episodes of cold to characterize the presence and stability of TM during the stress and growth responses of cold acclimation.
- Transcriptional memory mainly dampened stress responses as growth resumed and as *B. distachyon* became habituated to cold stress. Although permanent on vernalization gene *VRN1*, TMs were short‐term and reversible on cold‐stress genes. Growing under cold conditions also coincided with the acquisition of new and targeted cold‐induced transcriptional responses.
- Overall, TM provided plasticity to cold stress responses during cold acclimation in *B. distachyon*, leading to stress habituation, acquired stress responses, and resumed growth. Our study shows that chromatin‐associated TMs are involved in tuning plant responses to environmental change and, as such, regulate both stress and developmental components that characterize cold‐climate adaptation in *B. distachyon*.

## Introduction

Understanding the mechanisms of phenotypic plasticity in plants is crucial to building a sustainable agriculture, especially under the current world‐wide climate and environmental crises. Human activities have caused abrupt environmental changes that will affect the global climate well beyond this century, imposing stress of increasing intensity upon plants and crops (Gray & Brady, 2016; USGCRP, 2017; Bathiany *et al*., 2018). Environmental disruptions can be widely problematic; for example, cold spells and late frosts impair crop production in a wide variety of environments, from temperate Canada to sub‐tropical India (Aggarwal, 2008; Kutcher *et al*., 2010). Finding ways to employ or enhance the acclimation mechanisms of plants can contribute to building more resilient food production systems.

Stressful conditions generally trigger responses that alleviate the negative effects of stress on plant growth and development. Depending on the severity of the stress and the adaptability of the species, plants either die, enter a state of dormancy until conditions improve, or acclimate and continue growing (Ciarmiello *et al*., 2011). In the literature, acclimation is usually confined to reversible physiological changes, although stress conditions can irreversibly influence plant structure (Liu & Su, 2016; Klem *et al*., 2019; Mayer *et al*., 2020). Recently, stress responses were found to be particularly plastic, improving over multiple exposures (Ding *et al*., 2012; Li *et al*., 2019; Zuther *et al*., 2019; Mayer *et al*., 2020). These modified responses are influenced by stress exposures during which plants build ‘experience’ through stress memories (Crisp *et al*., 2016; Yeung *et al*., 2018).

Stress responses are often linked to extensive changes in gene expression (Ingram & Bartels, 1996; Thomashow, 1999; Zhang *et al*., 2006). Studies have hence reported cases of stress memories linked to transcription, called transcriptional memories (TMs). Genes that show TM typically display different transcriptional responses when the same stimulus is applied repeatedly (Avramova, 2015; Lämke & Bäurle, 2017). Transcriptional memory events were associated with specific epigenetic remodelers and to changes in chromatin marks left by stress exposure, which can, at least in part, explain the encoding of stress memories (Ding *et al*., 2012; Lamke *et al*., 2016). The di‐ and tri‐methylation of histone 3 lysine 4 (H3K4me2/3) were identified as markers of TM in various plant abiotic stress contexts, including heat, cold, drought and salt stress (Liu *et al*., 2014, 2018; Shen *et al*., 2014; Feng *et al*., 2016; Lamke *et al*., 2016; Zeng *et al*., 2019), while DNA methylation is involved in stress‐responses, TM and adaptation to environmental stress (Verhoeven *et al*., 2010; Jiang *et al*., 2014; Mayer *et al*., 2015; Sanchez & Paszkowski, 2014; Wibowo *et al*., 2016). By changing the regulation of gene expression, mechanisms of TM contribute to the plasticity of stress responses. As plants can progress from ‘shock‐like’ stress responses to resuming growth in stressful conditions, TM mechanisms may hence confer plasticity to such responses, leading to successful acclimation and adapted morphology. Although stress‐response mechanisms, in general terms, have been well studied, the function of TMs in this stress‐to‐growth requires further investigation.

The traits that characterize cold adaptation in the model cereal *Brachypodium distachyon*, namely cold acclimation and vernalization, provide a useful system for the study of the interaction between stress and growth, and the function of TM in acclimation. Recently established as a model for cold‐induced responses in temperate cereals, this undomesticated grass responds to cold by physiologically acclimating, gaining flowering competence through vernalization, and growing a cold‐hardy morphology (Li *et al*., 2012; Colton‐Gagnon *et al*., 2014; Ream *et al*., 2014; Ryu *et al*., 2014; Mayer *et al*., 2020). The acquisition of freezing tolerance, aimed at maximizing survival to winter, encompasses physiological changes associated with cold acclimation and morphological plasticity, leading to a freezing tolerant structure (Chouard, 1960; Thomashow, 1999; Körner, 2016). In addition, timely flowering, crucial in temperate climates, is ensured by a process known as vernalization. In temperate grasses, overwintering provides flowering competence by activating the transcription factor *VERNALIZATION1* (*VRN1*) (Danyluk *et al*., 2003; Oliver *et al*., 2009; Woods *et al*., 2017; Mayer *et al*., 2020). Under cold conditions, the chromatin state of *VRN1* becomes gradually depleted of the silencing epigenetic mark histone 3 lysine 27 trimethylation (H3K27me3), and this change remains after cold exposure (Oliver *et al*., 2009, 2013; Chen & Dubcovsky, 2012; Woods *et al*., 2017; Huan *et al*., 2018). Hence, vernalization ensures timely flowering over long‐term cold exposure through an epigenetically regulated permanent TM. Although well studied in vernalization, little is known about the function of TM in cold acclimation and morphological responses to cold. Recent work in Arabidopsis has demonstrated the existence of cold‐stress memories affecting freezing tolerance, and transcriptomic and metabolomic responses to chilling, and has highlighted the involvement of chromatin modifications in cold memory (Zuther *et al*., 2019; Vyse *et al*., 2020), though other studies have also described cold memory and changes to chromatin states associated with cold exposure other than vernalization (Zhu *et al*., 2008; Kwon *et al*., 2009; Mayer *et al*., 2015; Bittner *et al*., 2020). Unlike in Arabidopsis, however, vernalization and the expression of freezing tolerance encompassing cold acclimation and morphology are interconnected in *B. distachyon*, notably through *VRN1* which, other than regulating vernalization, influences cold acclimation and the acquisition of a winter‐hardy morphology (Bond *et al*., 2011; Mayer *et al*., 2020). Hence, studying the function of TM when growth is resumed in cold conditions in *B. distachyon* can provide valuable insights into the mechanisms that regulate phenotypic plasticity during stress acclimation in plants.

## Materials and Methods

### Plant growth and cold treatments

Bd21‐3 seeds were planted in 3 × 3 inch 0.5 l pots containing 160 g of G2 Agromix (Fafard et Frères Ltd, Saint‐Remi, QC, Canada), grown in an environmental growth chamber (Conviron, Winnipeg, MB, Canada) under nonstress control conditions (under a 16 h : 8 h, light : dark photoperiod at 22°C; 150 μmol m^−2^ s^−1^ photosynthetically active radiation (PAR) intensity) for 14 d, then transferred to 4°C or diurnal‐freezing conditions in an LT‐36VL growth chamber (Percival Scientific, Perry, IA, USA). The diurnal‐freezing treatment is characterized by air temperature cycles reaching −1°C at night and 22°C during the day, as described in Fig. 3(a) and as explained in more detail in Fig. 1(d) and (e) of Mayer *et al*. (2020). Plants were kept equally watered throughout.

### Measures of freezing tolerance

For whole‐plant freeze tests, plants were subjected to gradually decreasing temperatures from −1°C to −12°C at a rate of 1°C h^–1^ in a LT‐36VL chamber (Percival Scientific). Pots containing nine plants each were watered to soil saturation and placed randomly in the chamber. Three randomly selected pots were removed hourly as the temperature decreased from −8°C to −12°C, left to thaw at 4°C in the dark for 24 h, then switched to 22°C for an additional 24 h before being transferred to control conditions; percent survival was determined after 1 wk of recovery. This experiment was repeated three times. Electrolyte leakage for leaf tissue was measured as described in a study by Lee & Zhu (2010) for five leaf replicates, and was performed three times.

### RNA extraction and quantitative reverse transcription polymerase chain reaction (RT‐qPCR)

For each biological triplicate, a pool of aerial tissue from three plants was collected, flash‐frozen in liquid nitrogen, and extracted using an EZ‐10 RNA kit (cat. no. BS82314; Bio Basic, New York, NY, USA) following the manufacturer's protocol. Reverse‐transcriptase cDNA was obtained using iScript (cat. no. 1725037; Bio‐Rad) and was RT‐qPCR performed using Green‐2‐Go (cat. no. QPCR004; Bio Basic) and CFX Connect Real Time (BioRad), following the manufacturer's protocol. Relative transcript levels were determined by ΔΔCT using the *UBC18* gene as a reference for three biologically independent replicates (Hong *et al*., 2008; Mayer *et al*., 2020). The cold‐responsive genes selected have been studied previously and exhibit different functions during cold acclimation in *B. distachyon*. These include transcription factors *C‐REPEAT BINDING FACTOR1, 2 and 3* (*CBF1, CBF2, CBF3*) (Colton‐Gagnon *et al*., 2014; Ryu *et al*., 2014), which likely induce the expression of the following structural genes: membrane‐protein *COLD‐REGULATED413*, dehydrin *COLD‐REGULATED410* (Colton‐Gagnon *et al*., 2014; Mayer *et al*., 2020), and possibly the anti‐freeze protein *ICE RECRYSTALLIZATION INHIBITOR* (*IRI*) (Bredow *et al*., 2016; Mayer *et al*., 2020). The transcription factor *VERNALIZATION1* (*VRN1*) is involved in vernalization but was recently shown to play an important function in cold acclimation and freezing tolerance (Ream *et al*., 2014; Mayer *et al*., 2020). Primer sequences can be found in Supporting Information Table S1.

### Chromatin immunoprecipitation

Chromatin immunoprecipitation was performed for three biological replicates as previously described (Mayer *et al*., 2015), using antibodies anti‐Histone H3 (cat. no. ab1791; Abcam, Cambridge, UK), anti‐Histone H3 tri‐methyl‐K27 (cat. no. ab6002; Abcam), anti‐Histone H3 di‐methyl‐K4 (cat. no. ab11946; Abcam) and anti‐Histone H3 tri‐methyl‐K4 (cat. no. ab8580; Abcam). Immunoprecipitated samples were analyzed using qPCR and their percent input or percent H3 values were ascertained.

### Global DNA methylation assay

Global DNA methylation was performed using an Imprint Methylated‐DNA Quantification Kit (Sigma‐Aldrich) following the manufacturer's protocol. Genomic DNA was extracted using phenol‐chloroform for two independent biological replicates, each consisting of a pool of aerial tissue from three plants. Each replicate was measured in technical quadruplicate using a Microplate reader (Bio‐Rad).

### Sample preparation for RNA‐sequencing

For the diurnal‐freezing priming experiment, three plants for each treatment were collected 10 min before the light phase of the photoperiod. For the primed response to chilling experiment, three plants for each treatment were collected 3 h after the plants were moved to the chilling environment. After collection, plant tissue was flash‐frozen and stored at −80°C until extraction. This experiment was performed twice. RNA was extracted using the RNeasy Kit (Qiagen), following the manufacturer's protocol. Two libraries, composed of three biological replicates each, were built using NEBNext Multiplex Oligos for Illumina (cat. no. E7600S; New England Biolabs, Ipswich, MA, USA) and sequenced using a HiSeq 4000 (diurnal‐freezing priming experiment; Illumina, San Diego, CA, USA) and a NovaSeq 6000 (primed response to chilling; Illumina) at Le Centre d’Expertise et de Services Genome, Québec.

### Transcriptome analysis

Using galaxy, FASTQ reads (available online at https://www.ncbi.nlm.nih.gov/bioproject/PRJNA629906), were analysed with fastqc and processed with trimmomatic to remove adapters (Andrews, 2010; Bolger *et al*., 2014; Afgan *et al*., 2018). Using rna star, trimmed reads were mapped to Bd21v.3.1 obtained via Phytozome (*Brachypodium distachyon* Bd21v.3.1 DOE‐JGI, http://phytozome.jgi.doe.gov), and reads were counted using featurecounts (Vogel, 2010; Dobin *et al*., 2013; Liao *et al*., 2014). Using deseq2 with cold/priming and replicates as factors, fold‐change was calculated for S1/S0, S4/S0, S4/S1, and naïve and primed responses to chilling by comparing cold‐treated plants with their respective non‐stressed controls (Anders & Huber, 2010). Genes displaying significant differential expression (*P*‐adj < 0.05) and absolute fold change > 2 (FC > 2) in both replicates were selected for further analysis.

### Gene ontology (GO) enrichment analysis

Gene ontology analyses were performed using agrigo v.2.0 (Tian *et al*., 2017) with a false discovery rate (FDR) adjusted p‐value of 0.05 for genes with FC > 2, goseq for genes with FC > 4 (Young *et al*., 2010), and revigo (Supek *et al*., 2011) with associated over‐represented *P*‐adj values. The *Oryza sativa* GO term size database was used, along with simrel for semantic similarity (Supek *et al*., 2011). The annotated *B. distachyon* gene list was obtained from Phytozome. Gene ontology terms shown in Fig. 4(e) (see below) were obtained by summarizing agrigo terms with revigo. Treemaps were visualized using the package treemap in R (R Core Team, 2013).

### Transcription factors and transcriptional regulator analysis

Transcription factors were identified using itak (Zheng *et al*., 2016) and visualized with heatmap.2 in R (R Core Team, 2013).

### Statistical analyses

All experimental data, except for RNA‐seq results, were analyzed by one‐way ANOVA followed by Tukey's honest significance (HSD) test using jmp (SAS Institute, Cary, NC, USA). Statistical significance was determined with *P* < 0.05.

## Results

### Intermittent exposure to chilling resulted in a higher survival rate than continuous treatment

**Fig. 1:**
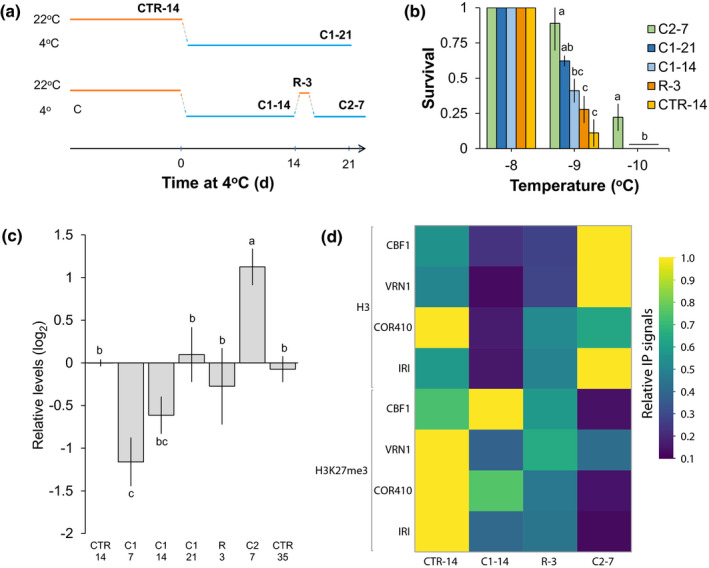
Repeated exposure to chilling leads to higher freezing tolerance, and to global and gene‐specific epigenetic changes. (a) Experimental design where one period of recovery at 22°C was inserted during cold acclimation under constant‐chilling (CC) at 4°C. C1, first exposure to chilling for 14 or 21 d (C1‐14 and C1‐21); C2‐7, plants re‐exposed to chilling for 7 d after recovery R‐3; CTR‐14, plants grown under control conditions at 22°C; R‐3, plants recovered from chilling for 3 d. (b) Survival rate under freezing conditions of CTR‐14, C1‐14, C1‐21, R‐3, and C2‐7 *Brachypodium distachyon* plants measured by whole‐plant freeze tests. (c) Relative global DNA‐methylation. C1, first exposure to chilling for 7, 14 or 21 d (C1‐7, C1‐14, C1‐21); C2‐7, second exposure to chilling for 7 d; CTR‐14, *B. distachyon* plants grown under control conditions at 22°C for 14 d; CTR‐35, *B. distachyon* plants grown under control conditions at 22°C for 35 d; R‐3, recovery from chilling at 22°C for 3 d. (d) Levels of histone 3 (H3) relative to input levels and of H3K27me3 relative to H3 of cold‐regulated genes *C‐REPEAT BINDING FACTOR1* (*CBF1*), *VERNALIZATION1* (*VRN1*), *COLD‐REGULATED410* (*COR410*) and *ICE RECRYSTALLIZATION INHIBITOR* (*IRI*) at CTR‐14, C1‐14, R‐3 and C2‐7. Different letters indicate statistical difference; *P* < 0.05; error bars represent SD between three biological replicates.

To determine whether *B. distachyon* can acquire cold‐stress memories, we exposed plants to a cold acclimation treatment interrupted by recovery in control conditions. By performing whole‐plant freeze tests, we measured survival under freezing conditions for plants that were exposed to chilling continuously for 14 or 21 d, or intermittently for 21 d (first for 14 d, then for 7 d separated by a 3‐d recovery; Fig. 1a). Plants exposed to chilling for a total of 21 d were most tolerant to freezing. However, those that were subjected to an intermittent treatment survived better than those exposed to continuous chilling (Fig. 1b). Interestingly, the second 7‐d exposure induced an increase in the 50% lethal temperature (LT_50_) value from *c.* 25% to *c.* 90% at − 9°C (re‐acclimated compared to recovery plants), compared to an increase from *c.* 15% to *c.* 40% after the first 14‐d exposure (acclimated compared to nonacclimated plants). Therefore, these results indicate that *B. distachyon* acclimated to cold faster and more efficiently during the second exposure to chilling.

### Repeated chilling led to global and gene specific chromatin changes

As chilling induces both cold acclimation and vernalization in *B. distachyon*, we investigated the chromatin response during both continuous and intermittent chilling treatments. We first measured the levels of global DNA methylation, which decreased significantly after 7 d of chilling but gradually increased back to control levels after 14 and 21 d of continuous exposure (Fig. 1c). Moving plants to recovery after 14 d in chilling did not alter the global levels of DNA methylation, while subsequent re‐exposure induced a hypermethylation significantly higher than the continuous 21‐d treatment (Fig. 1c). Therefore, continuous exposure to chilling led to a transient hypomethylation, while a second exposure to chilling led to global DNA hypermethylation.

As a depletion of the silencing mark H3K27me3 occurs during vernalization in *B. distachyon*, which is a long‐term epigenetic response, we investigated H3K27me3 levels on the gene *VERNALIZATION1* (*VRN1*), in acclimated, recovered and re‐acclimated plants. To determine whether this mark is also involved in cold acclimation, we measured H3K27me3 levels on the transcription factor *C‐REPEAT BINDING FACTOR1* (*CBF1*), the dehydrin *COR410*, and the anti‐freeze protein *ICE RECRYSTALLIZATION INHIBITOR* (*IRI*), which are involved in cold acclimation in *B. distachyon* (Colton‐Gagnon *et al*., 2014; Ryu *et al*., 2014; Bredow *et al*., 2016; Mayer *et al*., 2020). After 14 d of chilling, nucleosome levels significantly decreased on *VRN1*, *CBF1*, *COR410* and *IRI*, while levels of H3K27me3 generally decreased, suggesting that these genes were activated upon cold exposure (Fig. 1d; Table S2). Interestingly, 7 d of re‐acclimation led to even lower levels of H3K27me3, while nucleosome levels increased on the four genes. Therefore, cold acclimation led to fewer nucleosomes and lower levels of H3K27me3, while reacclimation led to denser chromatin, suggesting that *B. distachyon* had a different chromatin response upon re‐exposure to chilling.

### Cold acclimation induces the formation of TMs

**Fig. 2:**
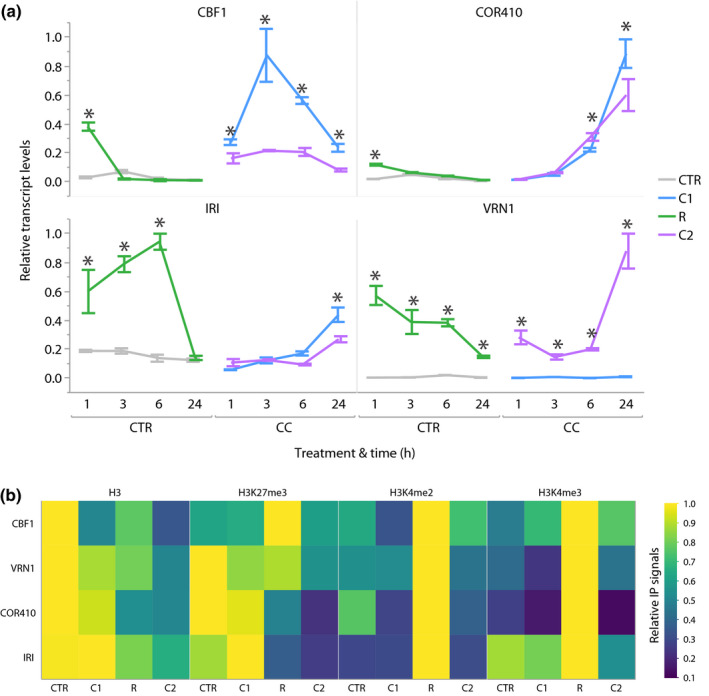
Repeated chilling influences transcriptional and chromatin responses to cold in *Brachypodium* *distachyon*. (a) Transcript levels of *COR* genes *CBF1, VRN1*, *COR410* and *IRI* at the first 1, 3, 6 and 24 h of exposure to a first cold treatment (C1), during recovery from chilling (R) and to a second cold treatment (C2) relative to CTR, C1‐14 and R‐3. (b) Levels of histone H3 relative to input levels, and of H3K27me3, H3K4me2 and H3K4me3 relative to H3 on *CBF1, VRN1*, *COR410* and *IRI* at 3 h into C1, R and C2 compared to control CTR. Asterisks indicate statistical difference (*, *P* < 0.05); error bars represent SD between three biological replicates.

The transcriptional response of cold acclimation typically occurs, unlike vernalization, during the first few h of cold exposure. Hence, to determine whether cold acclimation led to the formation of TMs, we measured the transcript levels of *CBF1*, *COR410, IRI* and *VRN1* at 1, 3, 6 and 24 h of exposure, during re‐exposure to chilling, and in recovery. The transcript levels of all four genes were measurably higher at least at the first h of recovery compared to nonacclimated controls (Fig. 2a). Interestingly, transcript levels of *IRI* increased within the first 6 h of recovery, while those of *VRN1*, known to be regulated by a permanent TM, remained elevated throughout recovery. Re‐exposure to chilling led to a lower transcriptional response at all time points in *CBF1* but only at the later ones in *COR410* and *IRI*, while *VRN1* showed higher activation at all time points (Fig. 2a). Therefore, cold exposure induced the formation of TMs that affected the transcript levels of *IRI* during recovery, of *CBF1* upon re‐exposure and of *VRN1* throughout.

As chilling led to the formation of TMs affecting *CBF1*, *COR410*, *IRI* and *VRN1*, we investigated whether these were connected, at their gene loci, to nucleosome occupancy, to levels of H3K27me3 and to the levels of chromatin marks involved in stress‐induced TMs, namely H3K4me2 and H3K4me3. These were measured during the early transcriptional response to cold acclimation at 3 h into the first and second exposures, and into recovery. The nucleosome and H3K27me3 levels measured under chilling were lower in plants exposed for a second time, indicating that these adopted a looser chromatin structure (Fig. 2b; Table S2). Moreover, the levels of H3K4me2 and H3K4me3 peaked at recovery, suggesting that these were deposited after the first episode of cold, and dropped to lower levels upon re‐exposure, which indicates that they are involved in the acquired transcriptional responses of *CBF1*, *VRN1 IRI* and *COR410* (Fig. 2b). Therefore, the TMs observed for *CBF1*, *COR410*, *IRI* and *VRN1* occurred along with different epigenetic signatures at their gene loci.

### Longer exposures to diurnal freezing translate into higher freezing tolerance

**Fig. 3:**
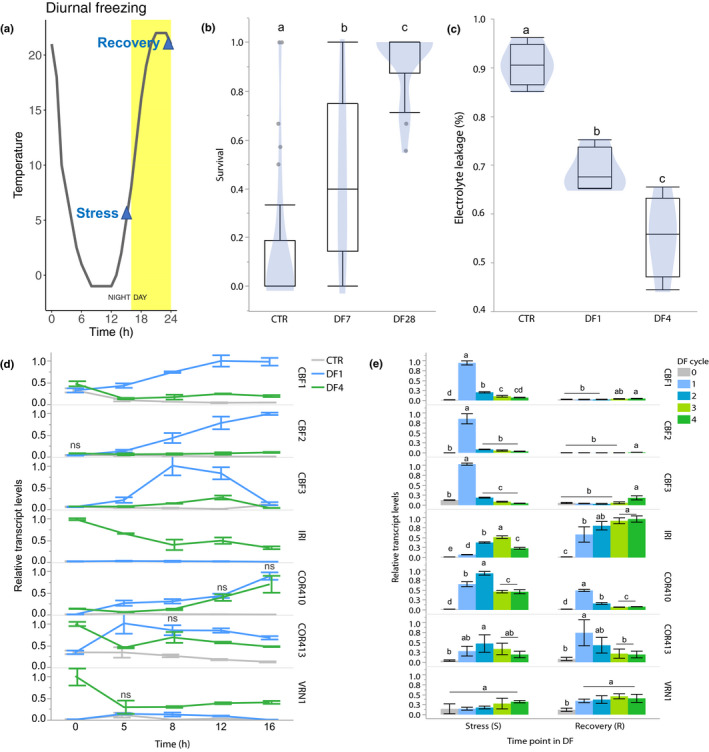
Cycles of diurnal freezing gradually increase freezing tolerance and change transcriptional responses to cold in *Brachypodium* *distachyon*. (a) One cycle of diurnal freezing, with time points S (stress) and R (recovery). (b) Freezing tolerance of plants exposed to 7 and 28 cycles of diurnal freezing (DF; DF7 and DF28), measured in whole‐plant freeze tests performed three times independently on 27 plants per temperature plateau between −8 and −12°C. (c) Damage induced by freezing of plants previously exposed to 1 or 4 cycles of DF (DF1 and DF4) measured by electrolyte leakage. (d) Relative transcript levels of *CBF*s, *IRI, COR410*, *COR413* and *VRN1* measured at 0, 5, 8, 12 and 16 of exposure to 1 or 4 cycles of DF (DF1 and DF4) relative to control (CTR). (e) Relative transcript level of *COR* genes in response to 1, 2, 3 and 4 cycles of DF (DF1–4) samples at S (S1–4) and R (R1–4) time points. Different letters indicate statistical difference; ns, no significant difference; *P* < 0.05. Error bars represent SD between three biological replicates.

We previously showed that chilling induces artificial responses and limits cold acclimation in *B. distachyon* partly through a high expression of *VRN1*, which inhibits the transcription of *CBF*s (Mayer *et al*., 2020). Hence, the high *VRN1* expression induced during the first chilling exposure has likely interfered with the transcriptional responses of re‐exposed plants. To address this limitation, we investigated cold acclimation under diurnal freezing, which models more closely the signals inducing cold acclimation and vernalization in the native range of *B. distachyon* (Mayer *et al*., 2020). Diurnal freezing is characterized by repeated 24‐h temperature cycles that fluctuate between day (22°C) and sub‐zero night temperatures (−1°C) under a 16 h : 8 h, light : dark photoperiod, and are therefore characterized by daily cycles of low‐temperature stress and recovery (Fig. 3a). Whole‐plant freeze tests performed on plants exposed to 7 and 28 cycles of diurnal freezing indicate that the treatment induces a gradually higher tolerance (Fig. 3b). In addition, while whole‐plant freeze‐tests also indicate this trend in plants exposed to 1 and 4 cycles of diurnal freezing (Fig. S1), electrolyte leakage assays show that plants exposed to 4 cycles were less damaged by freezing than plants exposed to only 1 cycle of diurnal freezing, which were both less damaged than nontreated control plants (Fig. 3b). Therefore, the freezing tolerance of *B. distachyon* increased over repeated cycles of diurnal freezing, indicating that these gradually prime *B. distachyon* to survive freezing (Fig. 3b,c).

### Transcriptional responses evolve over cycles of diurnal freezing

To determine whether cold‐induced transcriptional responses change over time in diurnal freezing, we measured the transcript levels of *CBF1*, *COR410*, *IRI* and *VRN1*. In addition, to deepen our analysis, the study of additional genes involved in cold acclimation in the species, namely the transcription factors *CBF2* and *CBF3* and the structural gene *COR413*, were also included. The transcript levels of the seven genes were measured at 0, 5, 8, 12 and 16 h of exposure to low‐temperature conditions during a first and a fourth cycle of diurnal freezing (Fig. 3a,d). Except for *IRI*, all transcripts accumulated within 16 h of exposure to the first cycle. During the fourth cycle, transcript levels were higher for *IRI* and *VRN1*, and lower for *CBF*s compared to the first, indicating that repeated cycles of diurnal freezing led to the establishment of TMs on these genes.

To investigate the changing transcript levels under diurnal freezing, we selected two time points: a stress time point which showed the highest difference in transcript levels from cycle 1 to cycle 4 (time point = 16 in Fig. 3d), and a recovery time point (time point = 0 or 24 in Fig. 3d). Transcript levels of *CBF*s decreased sharply after cycle 1 at the stress time point, but slowly increased from cycle 1 to 4 at recovery (Fig. 3d). The transcript levels of *IRI* gradually increased from cycle 1 to 4, especially at recovery, while the transcriptional responses of *COR410* and *COR413* remained fairly similar at stress time points, but decreased at recovery (Fig. 3d). *VRN1* transcripts gradually increased from cycle 1 to 4 at both stress and recovery time points (Fig. 3d). Therefore, all genes showed altered transcriptional responses to cycles of diurnal freezing. *CBF*s and *COR410–413* showed clear signs of TM that decreased their expression during stress and recovery, respectively, while *IRI* showed a TM that increased its transcriptional response at recovery.

### Six profiles describe the evolution of transcriptional responses to diurnal freezing

**Fig. 4:**
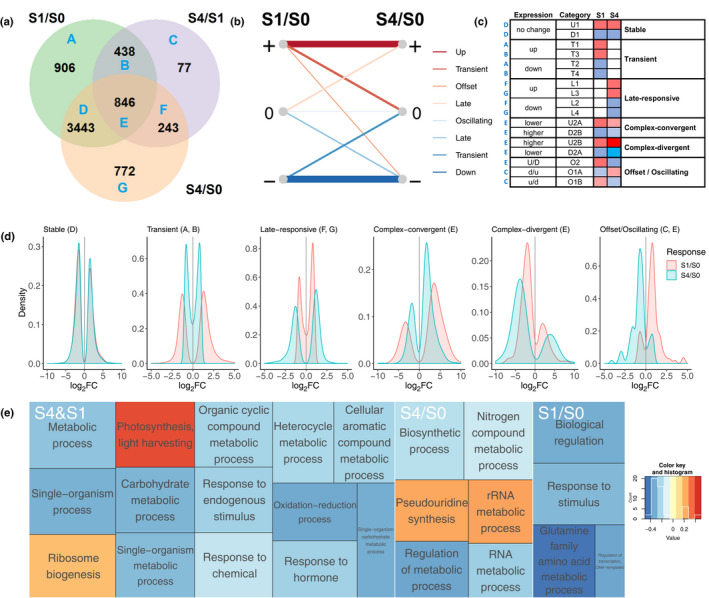
Transcriptional responses of *Brachypodium distachyon* evolve through repeated cycles of diurnal freezing following six profiles. (a) Venn diagrams of significantly differentially‐expressed genes in S1 (sampled during a first cycle of diurnal freezing) compared to S0 (control conditions), labelled S1/S0; in S4 (sampled during a fourth cycle of diurnal freezing) compared to S1, labelled S4/S1; and in S4 compared to S0, labelled S4/S0. (b) Sign of differential expression of genes responsive to diurnal freezing in S1/S0 and S4/S0. +, upregulated; 0, nonresponsive; −, downregulated. Lines are proportional to the number of genes following each trend. (c) Genes responsive to diurnal freezing divided into 17 categories according to their distribution between S1/S0, S4/S1 and S4/S0, as illustrated in (a), and their upregulation or downregulation in S1 and S4 responses (S1/S0 and S4/S0) as illustrated in (b). (d) Six profiles that describe the transcriptional behavior of genes under diurnal freezing. (e) Significantly over‐represented GO terms that are unique to the S1 response (S1/S0), unique to the S4 response (S4/S0), or shared by both (S4&S1). Colours depict the transcriptional change of genes associated with each GO term between S1 and S4 (S4/S1 – FC negative to positive, blue to red).

To characterize the transcriptional change induced by diurnal freezing, we compared the transcriptomes of plants exposed to the stress time point of cycle 1 (S1), or of cycle 4 (S4) of diurnal freezing, to those of nontreated controls (S0; Fig. 3a). A total of 6725 genes showed an FC > 2 when comparing at least one condition to another: cycle 1 of diurnal freezing compared to control (S1 compared to S0; S1/S0), cycle 4 of diurnal freezing compared to control (S4 compared to S0; S4/S0), or cycle 4 of diurnal freezing compared to cycle 1 (S4 compared to S1; S4/S1), which can be summarized using a Venn diagram, as shown in Fig. 4(a). We further classified these genes depending on whether they were upregulated, downregulated or nonresponsive in response to cold under diurnal freezing conditions at S1 and S4 (S1/S0 and S4/S0; Fig. 4b). Combining the categories of the Venn diagram and the genes’ responses in S1 and S4, we obtained a total of 17 distinct categories, which describe in detail the transcript level outcomes in plants responding to diurnal freezing (Table 1; Fig. 4c). These could be further regrouped into six expression profiles (Fig. 4c,d): *stable* genes showed no change in response to diurnal freezing, *transient* genes responded only in S1/S0, while *late‐responsive* genes responded only in S4/S0. *Complex‐convergent* and *complex‐divergent* genes responded in both S1/S0 and S4/S0, but *complex‐convergent* had lower expression in S4/S0 compared to S1/S0, and *complex‐divergent* had increased expression between S4/S0 and S1/S0. *Offset/oscillating* moved from upregulated to downregulated (offset with FC > 2, oscillating with FC < 2) or from downregulated to upregulated (oscillating FC < 2) between S1/S0 and S4/S0. Hence, these six expression profiles describe the main outcomes of transcriptional change between the first and the fourth cycles of diurnal freezing (Table 1; Fig 4c,d).

**Table 1:** Distribution of diurnal‐freezing responsive genes of *Brachypodium distachyon* according to fold change value for S1 and S4 responses, and according to the change in expression between S1 and S4.

			Venn diagram groups											
			A	B	C	D	E	F	G					
		S1/S0	FC > 2	FC > 2		FC > 2	FC > 2							
		S4/S0				FC > 2	FC > 2	FC > 2	FC > 2					
		S1/S4		FC > 2	FC > 2		FC > 2	FC > 2						
**Trend relative to control**			**Transient**		**Oscillating**	**Stable**	**Complex**	**Late responsive**						
**S1**	**S4**	**S4–S1**	**A**	B*	**C**	**D**	E*	**F**	**G**	**Total**	**%**	**Group**	**Transcriptional profiles**	
UP 2851 (42%)	UP 2063 (31%)	+					72 (1%)			72	(1%)	U2B	Complex‐divergent	72
		=				1600 (24%)				1600	(23%)	U1	Stable	1600
		−					391 (6%)			391	(5%)	U2A	Complex‐convergent	391
	=	−	444 (7%)	314 (5%)						758	(11%)	T1–T3	Transient	444–314
	DOWN	−					30 (0.4%)			30	(0.4%)	O2	Offset	30
= 1092 (16%)	UP	+						69 (1%)	461 (7%)	530	(7%)	L1–L3	Late response	69–461
	=	+			16 (0.2%)					16	(0.2%)	O1A	Oscillating	16
		−			61 (0.9%)					61	(0.9%)	O1B	Oscillating	61
	DOWN	−						174 (2%)	311 (5%)	485	(7%)	L2–L4	Late response	174–311
DOWN 2782 (41%)	=	+	462 (7%)	124 (2%)						586	(8%)	T2–T4	Transient	462–124
	DOWN (33%)	+					163 (2%)			163	(2%)	D2B	Complex‐convergent	163
		=				1843 (27%)				1843	(27%)	D1	Stable	1843
		−					190 (3%)			190	(2%)	D2A	Complex‐divergent	190
		Total	906 (13%)	438 (7%)	77 (1%)	3443 (51%)	846 (13%)	243 (4%)	772 (11%)	6725				

### The progression from S1 to S4 responses suggests a transition from stress to growth in diurnal freezing

The response to cycle 1 of diurnal freezing (S1 response) and cycle 4 of diurnal freezing (S4 response) shared respectively 61% and 65% of their transcriptomes (the same transcripts in the same amount, corresponding to *stable* genes). In addition, 15% and 16% of their transcriptomes contained the same transcripts, but in different amounts (corresponding to *complex‐convergent*, *complex‐divergent* and *offset/oscillating* genes), while unique transcripts corresponded to 24% and 19% of their transcriptomes respectively (*transient* or *late‐responsive* genes; Table 2). To characterize the progression from S1 to S4, enriched gene ontology terms shared between, and unique to, S1 and S4 were visualized along with the average S4/S1 fold‐change of associated genes (Figs. 4e, S2, S3). Photosynthesis and ribosome biogenesis genes were mostly upregulated, while oxidation/reduction and metabolism genes were mostly downregulated between S1 and S4 (Fig. 4e). Further analysis revealed that transcription factor activity and responses related to oxygen and heme binding were significantly enriched in S1 and S4 responses (Table 3). Interestingly, *iron ion binding*, *response to auxin*, *photosynthesis* and *sequence‐specific DNA binding* were enriched in S1, but not in S4, indicating that S1 partly included transient responses. Specifically, the absence of the term *photosynthesis* in S4 and the upregulation of associated genes between S1 and S4 suggest that the normal expression of photosynthesis was restored in S4 (Fig. 4e; Table 3). Hence, the response to diurnal freezing was plastic, and clearly changed between S1 and S4. Priest *et al*. (2014) described 22 distinct gene modules that characterize the plasticity of the abiotic stress response in *B. distachyon*. A comparison between the diurnal‐freezing responsive genes and these modules supports the idea that S1 was associated with transiently expressed transcription factors and S4 with growth‐related responses and restored photosynthesis (Fig. S4). Therefore, repeated cycles of diurnal freezing led to extensive reorganization of cold‐induced responses from S1 to S4, indicating that *B. distachyon* exhibited plastic responses during cold acclimation.

**Table 2:** Distribution of common and unique genes between S1 and S4 responses in *Brachypodium distachyon*.

	S1	S4
Responsive	5633 (84%)	5304 (79%)
Up	2851	2756
Down	2782	2548
Shared	4289 (76%)	4289 (81%)
Similar levels	3443 (61%)	(65%)
Different levels	846 (15%)	(16%)
Unique	1344 (24%)	1015 (19%)
Unresponsive	1092 (16%)	1421 (21%)
Total	6725	

**Table 3:** Gene ontology enrichment analysis in diurnal freezing responsive genes (DFRG) and their distribution in S1 and S4 responses in of *Brachypodium distachyon* using GOseq.

	GO term		DFRG				S1				S4			
		Ontol.	DE	Tot	p.adj.or	p.adj.ur	DE	Tot	p.adj.or	p.adj.ur	DE	Tot	p.adj.or	p.adj.ur
S1&S4	Transcription factor activity, sequence‐specific DNA binding	MF	164	364	8.01E‐08	1	81	364	2.99E‐11	1	61	364	4.26E‐06	1
S1&S4	Regulation of transcription, DNA‐templated	BP	284	707	8.01E‐08	1	109	707	1.17E‐06	1	89	707	7.43E‐05	1
S1&S4	Oxidoreductase activity, acting on paired donors…*	MF	71	150	0.00095	1	31	150	0.00064	1	25	150	0.01005	1
S1&S4	Heme binding	MF	108	249	0.00046	1	43	249	0.00419	1	37	249	0.01005	1
S1	Iron ion binding	MF	76	180	0.02655	1	33	180	0.0041	1	26	180	0.06271	1
S1	Response to auxin	BP	22	34	0.00521	1	12	34	0.03424	1	10	34	0.13828	1
S1	Photosynthesis, light harvesting	BP	15	18	0.00095	1	12	18	5.85E‐06	1	7	18	0.06271	1
S1	Sequence‐specific DNA binding	MF	103	215	2.86E‐06	1	41	215	0.00127	1	30	215	0.11307	1
DFRG	Calmodulin binding	MF	18	26	0.00488	1	3	26	1	1	2	26	1	1
DFRG	Response to stress	BP	35	67	0.01228	1	15	67	0.12457	1	13	67	0.17717	1
DFRG	Oxidation‐reduction process	BP	291	812	0.00654	1	94	812	0.23589	1	81	812	0.18156	1
S1	Carboxylic acid metabolic process	BP	13	25	0.72934	1	9	25	0.02881	1	7	25	0.17717	1
S4	Trehalose biosynthetic process	BP	12	17	0.05087	1	4	17	1	1	8	17	0.00071	1
S1&S4	Structural constituent of ribosome	MF	49	247	1	0.0299	0	247	1	0	1	247	1	0
S1&S4	GTP binding	MF	24	155	1	0.0082	2	155	1	0.0179	0	155	1	0.00177
S1&S4	Ribosome	CC	47	239	1	0.0299	0	239	1	0	1	239	1	2.52E‐07
S1&S4	Translation	BP	51	256	1	0.0299	0	256	1	0	1	256	1	0

*p.adj.or* adjusted *P*‐value for over‐representation.

*p.adj.ur* adjusted *P*‐value for under‐representation.

* …with incorporation or reduction of molecular oxygen.

### Transcriptional memories regulate transient stress responses

To characterize the transcriptional regulation that accompanied the response plasticity in diurnal freezing, we investigated the distribution of transcription factors within the six expression profiles identified in Fig. 4(c,d). *Transient* and *complex‐convergent* expression profiles were especially enriched in transcription factors, while the *stable* profile regrouped the most (Table 4). Interestingly, there were twice as many transcription factors in the *transient* than there were in the *late‐responsive* expression profile. Hence, S1 regrouped more transcription factors both in terms of proportion and absolute number compared to S4. Moreover, *transient* transcription factors function mostly in stress responses, *late‐responsive* in growth and chromatin remodeling, and *complex‐convergent* in both stress and development‐related factors (Table 4). Hence, as suggested by the previous gene ontology analyses, the response to diurnal freezing generally progressed from the expression of stress‐related to growth‐related transcriptional responses.

**Table 4:** Distribution of transcription factors in the six expression profiles of diurnal‐freezing responsive genes in *Brachypodium distachyon*.

Number of transcription factors and regulators												
DFRG profile		%	Category		%	S1	%	S4	%	Representative	General function	References
Transient	128	10	T1	41	9	128	26	0		C2H2	Stress response	Wang *et al*. (2019)
			T2	41	9					E2F‐DP	Cell cycle, cell growth	Ramirez‐Parra *et al*. (2018)
			T3	36	11					GeBP	Cytokinin signalling	Chevalier *et al*. (2008)
			T4	10	8					HMG	Stress response, growth	Kwak *et al*. (2007)
										IWS1	Brassinosteroid‐responsive	Belkhadir & Jaillais (2015)
												Li *et al*. (2010)
										MBF1	Stress response, development	Jaimes‐Miranda & Chávez Montes (2020)
										Tify	Stress response, development	Zhang *et al*. (2015)
												
Stable	278	8	U1	126	8	361	73	361	83			
			D1	152	8							
Complex‐convergent	61	11	D2B	16	10					AP2/ERF	Stress response, hormone and development	Xie *et al*. (2019)
			U2A	45	12					LDB	Plant growth and development	Fan *et al*. (2012)
										WRKY	Stress response	Phukan *et al*. (2016)
												
Complex‐divergent	22	8	D2A	16	8							
			U2B	6	8							
Late response	64	6	L1	13	19	0		64	15	Alfin‐like	Chromatin modifier	Sanchez & Zhou (2011)
			L2	13	7					BES1	Plant growth from multiple signals	Li *et al*. (2018)
			L3	18	4					PHD	Chromatin modifier	Sanchez & Zhou (2012)
			L4	20	6					SNF2	Chromatin remodelling, may modulate stress‐growth	Mlynárová *et al*. (2007)
Offset/Oscillating	7	7	O2	2	7	7	1	7	2			
			O1A	3	19							
			O1B	4	7							
Total DFRG	542	8										

Genes responsive in S1 and which showed significant changes in expression between S4 and S1 (i.e. *transient* T3 and T4 and *complex* genes), were regulated by TMs (Fig. 4a,d; Table 1). Hence, memory genes responded differently between the first and the fourth cycle of diurnal freezing, which was validated by RT‐qPCR analysis (Fig. S5). Accounting for 19% of the diurnal‐freezing responsive genes, memory genes were enriched in transcription factors generally involved in the stress response. For example, most members of the AP2/ERF family were regulated by TMs (Fig. S6). This analysis indicated that *CBF1* and *CBF2* were *complex‐convergent*, *IRI* and *CBF3* were *complex‐divergent* and *COR410* and *COR413* were *stable* genes. *VRN1* behaved like *complex‐divergent* genes (but with lower fold‐change). Hence, RNA‐seq data support our previous results that *CBF*s*, IRI* and *VRN1* displayed TM.

### The TMs of genes involved in cold acclimation are mostly reversible

**Fig. 5:**
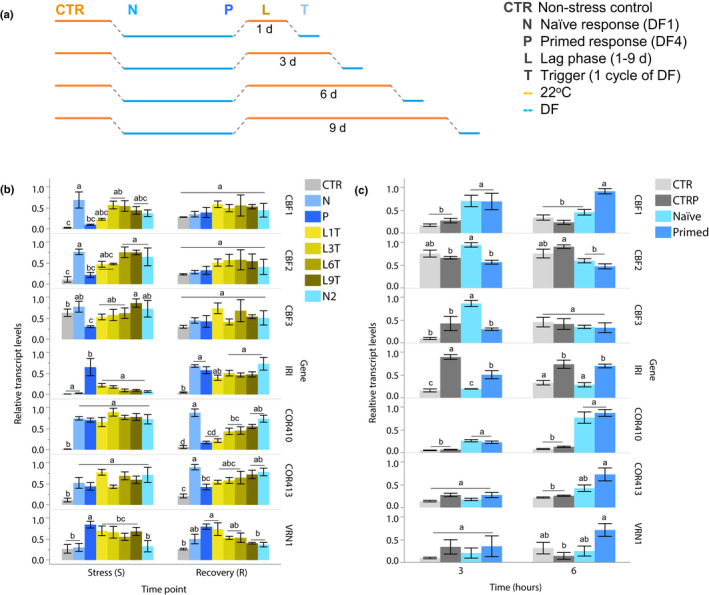
Priming induced by diurnal freezing leads to reversible transcriptional memories on cold‐responsive genes in *Brachypodium* *distachyon*. (a) Diagram of the experimental design, showing the naïve (N) and primed (P) responses, lag phase (L), and a trigger cycle (T). (b) Naïve (N), primed (P), trigger responses after 1, 3, 6 and 9 d of lag (L1‐9T), and naïve age control (N2). (c) Transcriptional levels of cold‐responsive genes in response to chilling in primed and naïve plants compared to primed control (CTRP) and naïve control (CTR). Different letters indicate statistical difference; *P* < 0.05; error bars represent SD between three biological replicates.

Plants exposed to diurnal freezing became increasingly tolerant to freezing and gained TMs. Hence, *B. distachyon* became primed to respond to freezing, with the response to cycle 1 of diurnal freezing considered naïve, and the response to cycle 4 considered primed. To determine the stability of TMs on genes involved in cold acclimation, primed plants (exposed to four cycles of diurnal freezing) were exposed to an extended recovery (lag phase) for 1, 3, 6 or 9 d at 22°C, then to a trigger cycle of diurnal freezing (Fig. 5a). Measured at the stress time point of diurnal freezing, the response of *COR410* and *COR413* was naïve throughout, while the *VRN1* response remained primed after all lag times (Fig. 5b). Moreover, the transcriptional responses of *CBF1*, *CBF2*, *CBF3* and *IRI* gradually returned to their naïve states after 3–6 d of lag. Therefore, the TM was stable for *VRN1* but reversible for *CBF1*, *CBF2*, *CBF3* and *IRI*. Interestingly, all seven genes responded differently to priming at the recovery time point (Fig. 5b). For instance, the transcriptional responses of *CBF1*, *CBF2*, *CBF3* and *IRI* were naïve at all stages, hence showing no signs of memory at recovery, while *VRN1*, *COR410* and *COR413* showed reversible memories. Overall, diurnal freezing induced TMs in primed plants that were mostly reversible after 3–6 d under control conditions.

### Primed plants respond less intensely to chilling and retained the responses acquired in diurnal freezing

Genes involved in cold acclimation demonstrated different types of TMs at the stress and recovery time points of diurnal freezing, which could indicate that temperature cycles influenced their expression, for example inducing a change in circadian regulation. To address this possibility and confirm the presence of TM, we applied cold at an unexpected time (during the middle of the day as opposed to dusk/night as in diurnal freezing) and measured the transcriptional response of primed plants exposed to chilling after one lag‐cycle (when memories had not reverted to the naïve state) compared to naïve plants exposed to chilling, and to the nonstressed primed and naïve control plants (Fig. 5c). In primed plants, *CBF1* showed a more sustained response at 6 h, while *CBF2* and *CBF3* responded with lower levels at 3 h compared to naïve plants (Fig. 5c). Moreover, *IRI* levels were higher in primed plants both in chilling and control conditions, confirming that *IRI* acquired a strong TM, while *COR410* and *COR413* showed no difference in expression after priming. As expected, *VRN1* transcripts accumulated to higher levels in primed plants, which have likely undergone early vernalization (Fig. 5b). Therefore, primed plants exhibited TMs formed under diurnal freezing conditions when exposed to chilling.

**Fig. 6:**
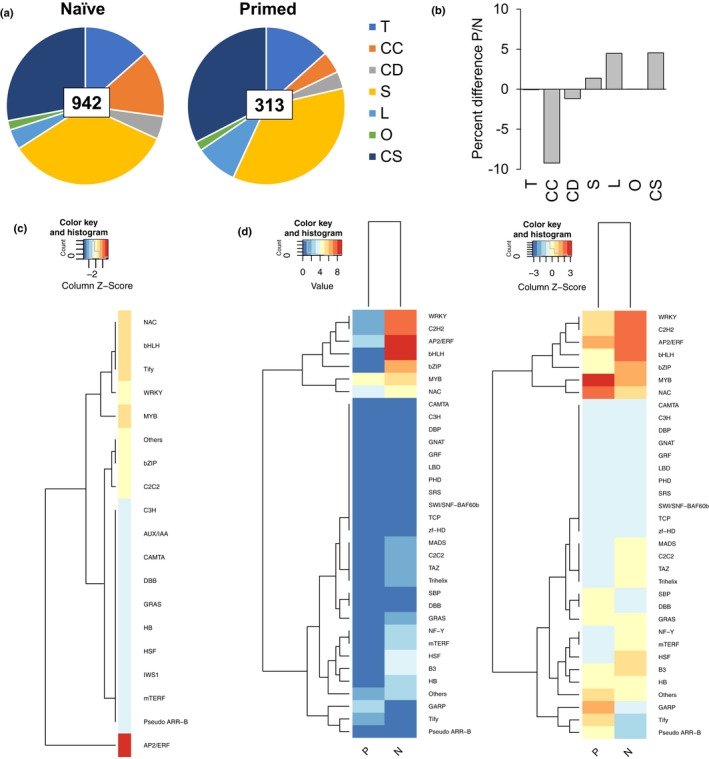
Primed *Brachypodium* *distachyon* acquired an attenuated response to chilling. (a) Proportion of chilling‐responsive genes unique to naïve (942 genes) or primed responses (313 genes) that belong to the expression profiles transient (T), complex‐convergent (CC), complex‐divergent (CD), stable (S), late‐response (L) and offset/oscillating (O) or not found to be responsive under diurnal‐freezing (chilling‐specific; CS). (b) Difference between the distribution of genes in diurnal‐freezing expression profiles and the chilling‐specific category in naïve (N) or in primed response (P). (c) Transcription factors and transcriptional regulators that are responsive in both N and P. (d) Number of transcription factors and transcriptional regulators found in genes unique to N and P in absolute value (left) and relative to the total number of genes in P and N (right).

Furthermore, we compared the transcriptome of naïve and primed plants at 3 h of exposure to chilling to nonstressed controls. The primed response consisted of approximately half the number of responsive genes observed for the naïve response (Fig. 6a; Table 5). 412 genes showed no memory, while 942 were altered by TMs – a result that was validated by RT‐qPCR analysis (Table 5; Fig. S7). All six expression profiles identified in diurnal freezing were represented in the naïve and primed responses to chilling. The primed response had a 3‐fold depletion in *complex‐convergent* genes and a 2.3‐fold enrichment in *late‐responsive* genes compared to the naïve response, indicating that the responses acquired in diurnal freezing were retained in primed plants (Fig. 6a; Table 5). Diurnal freezing led to TMs on all naïve‐specific genes (unique to the naïve response), and on genes that showed a different response in naïve and primed plants. Over 70% of the naïve response consisted of memory genes.

**Table 5:** Transcriptional behavior of chilling‐responsive genes that fit into diurnal‐freezing expression profiles and that show transcriptional memory in *Brachypodium distachyon*.

	N & P same response (FC < 2)	N & P different response (FC > 2)	Unique to N –nonresponsive in P	Response in P only	Total	%
DF‐responsive profiles						
Transient	76	7	127	42	252	15
Complex‐convergent	107	16	129	14	266	15
Complex‐divergent	9	3	44	11	67	4
Stable	131	17	321	111	580	34
Late response	17	1	39	27	84	5
Offset/Oscillating	7	0	18	6	32	2
Chilling‐specific	64	6	264	102	436	25
Total	412	50	942	313	1717	100
% Total	24	3	55	18		
% Naive	29	4	67	–		
% Primed	53	6	–	40		
DF‐responsive						
Memory (M)	174 (50%)	27 (59%)	249 (37%)	44 (21%)	494	30
No memory (NM)	173 (50%)	18 (41%)	429 (63%)	167 (79%)	787	50
Ratio M/NM	1	1.5	0.6	0.3	0.6	
TF/TR	72	6	96	33		
In response to CC	Stable	Memory, complex	Memory, silenced	Acquired response		
Genes showing memory overall	174	50	942	44	1210	70

### The primed response to chilling is depleted in transcription factors and enriched in structural genes

Naïve‐specific genes were enriched in *nucleic acid binding* and *sequence‐specific DNA binding transcription factor activity* whereas primed‐specific genes were *enriched in metabolic, oxido‐reduction, oxoacid, organic acid and cellular processes* (Table S3). Although the number of primed‐specific genes was three times lower than the number of naïve‐specific genes, the primed‐specific genes were associated with four times the number of enriched gene ontology terms, and hence were connected to a wider diversity of functions, related to plant metabolism and cellular processes, as opposed to naïve‐specific genes, whose function is mostly related to transcriptional regulation. Among the genes that were differently expressed between the naïve and primed responses, 66% were dampened by diurnal freezing (Table S4). Of these genes, which became hyposensitive to cold, many were stress‐response transcription factors (Tables S4, S5). Indeed, the primed response contained fewer stress‐response transcription factors of the families AP2/ERF, bHLH, WRKY and C2C2 (Figs 6c,d, S8). Overall, the primed response to chilling was mostly depleted in stress‐response transcription factors and contained a higher proportion of responsive structural genes.

### Repeated priming in diurnal freezing led to the formation of similar TMs but a different chromatin response

**Fig. 7:**
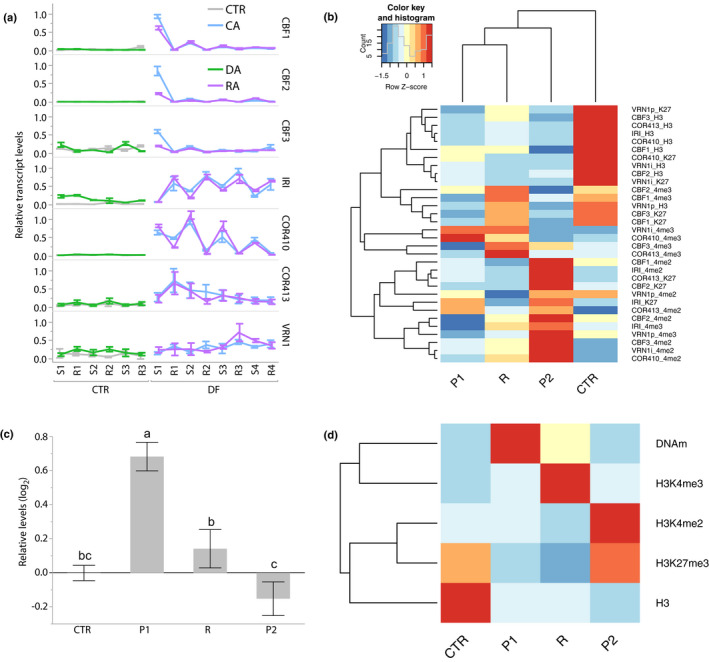
Repetitive priming under diurnal freezing (DF) induced similar effects on transcription but distinct chromatin responses in *Brachypodium distachyon*. (a) Relative transcript levels during a first (P1) and second (P2) episode of priming under DF separated by a 3‐d recovery from DF (R) in COR genes *CBF*s, *IRI*, *COR410–413* and *VRN1*. (b) Relative levels of epigenetic marks H3, H3K27me3 (k27), H3K4me2 (4me2) and H3K4me3 (4me3) in CTR, P1, R and P2. (c) Relative global levels of DNA methylation in CTR, P1, R and P2. (d) Summary of the epigenetic change on *COR* genes and global DNA methylation levels during repeated priming. Different letters indicate statistical difference; *P* < 0.05; error bars represent SD between three biological replicates.

To investigate whether a second episode of priming could reinforce TMs and induce chromatin changes, we compared the transcriptional response of genes involved in cold acclimation and their associated levels of H3, H3K27me3, H3K4me2, H3K4me3, and the levels of global DNA methylation, to two episodes of priming in diurnal freezing separated by 3‐d recovery. The changes in the transcriptional responses to diurnal freezing were similar for both priming episodes (Fig. 7a). However, the first and second episodes of priming led to distinct chromatin signatures. Genes became generally depleted in H3 and H3K27me3 after the first priming episode, and this effect remained at recovery, along with increased levels of H3K4me3. During the second episode of priming, genes also became generally depleted in H3K4me3, but enriched in H3K4me2 (Figs S9, 7b). Global DNA methylation increased at the first priming episode, but decreased over recovery and during the second episode of priming, indicating contrasting chromatin responses to diurnal freezing (Fig. 7c). Overall, the chromatin composition of genes involved in cold acclimation evolved in response to repeated episodes of priming in diurnal freezing without affecting the formation of TMs, suggesting that chromatin composition can evolve separately from transcriptional responses.

## Discussion

### Cold acclimation in *B. distachyon* is characterized by a transition from stress to growth responses

*Brachypodium distachyon* gradually acquired higher freezing‐tolerance under diurnal‐freezing conditions (Fig. 3b,c). This treatment first induced transcriptional responses typical of cold acclimation, including the activation of the transcription factors, *C‐repeat binding factors* (*CBF*s), and structural genes *IRI*, *COR410* and *COR413* (Fig. 3d). Over repetitions of diurnal‐freezing cycles, the transcriptional responses associated with cold acclimation were dampened, while the initial downregulation of photosynthesis, which is a typical sign of stress, returned to control levels (Figs 3d–e, 4e, 6; Tables 3, 4). As plants grown under diurnal‐freezing conditions eventually produce biomass equivalent to nonstressed control plants, *B. distachyon* could hence successfully acclimate to the treatment (Crisp *et al*., 2016; Mayer *et al*., 2020). Interestingly, almost 40% of the cold‐response transcriptome changed at the fourth cycle of diurnal freezing, where initial stress response genes were generally replaced by others linked to primary metabolic processes, and cellular growth and development (Figs 4, S2–3; Table 4). Furthermore, responsive genes were enriched in iron binding, heme binding and oxygen signaling genes. Plant hemoglobins are important for normal plant growth (Hebelstrup *et al*., 2006), and by regulating the interplay between oxygen and nitric oxide, they mediate stress and growth responses to environmental changes (Gupta *et al*., 2011; Simontacchi *et al*., 2015). Therefore, these results indicate that *B. distachyon* readjusted stress responses, growth and cellular homeostasis over time under diurnal‐freezing conditions (Table 3). As this treatment was designed to reproduce the natural signals of cold acclimation in *B. distachyon* (Mayer *et al*., 2020), the stress‐to‐growth transition indicates that morphology may be an extension of cold acclimation in the species.

The transiently high expression of *CBF*s, important regulators of cold acclimation in several plant species, is likely important for morphological cold acclimation in *B. distachyon*. Well studied in cold acclimation, *CBF*s are known regulators of structural cold‐regulated genes’ expression (Jaglo‐Ottensen *et al*., 1998). In response to repeated chilling and diurnal‐freezing, the initial transcriptional responses of *CBF*s are dampened (Figs 2a, 3d, 5b). *C‐repeat binding factors* induce the expression of *COR* genes by binding to the C‐repeat motif in their promoter (Thomashow, 1999), but this has yet to be determined experimentally in *B. distachyon*. Here, results indicate that the maintenance of *COR410/413* expression post‐cold is correlated to the TM status of *CBF*s, suggesting the existence of a *CBF/COR* regulatory link in *B. distachyon* (Figs 5b,c, S10). Furthermore, *CBF1*, *CBF2* and *CBF3* have redundant and essential cold‐acclimating functions in Arabidopsis, confer freezing tolerance in many plant species, and are involved in other processes, such as seedling and chloroplast development (Gilmour *et al*., 2004; Savitch *et al*., 2005; Benedict *et al*., 2006; Jia *et al*., 2016; Zhao *et al*., 2016). Their regulation receives inputs from the circadian clock, light, and temperature for an appropriate response (Nakamichi *et al*., 2009; Jiang *et al*., 2017; Liu *et al*., 2017). The constitutive overexpression of *CBF*s usually provides high freezing tolerance but severely limits plant growth and delays development in many plant species, notably through the accumulation of DELLA proteins (Achard *et al*., 2008; Jeknic *et al*., 2014; Wisniewski *et al*., 2015). Therefore, the high expression of *CBF*s under diurnal‐freezing conditions may be transient to allow *B. distachyon* to grow.

### Cold acclimation in *B. distachyon* relies on a habituation response mediated by TMs

Cold acclimation and the increase in freezing tolerance coincided with the establishment of TMs. These mainly dampened initial cold‐stress transcriptional responses (Figs 2a, 3d,e). Approximately 20% of diurnal‐freezing responsive genes showed strong TMs, with 75% of these leading to downregulation (Table 1). Moreover, over 67% of naïve‐response genes became nonresponsive under chilling in primed plants, revealing that most TMs were silencing/downregulating memories (Table 5). Generally, studies report memory genes that show stronger and faster responses (i.e. showing hyperactivation) under repeated exposure to a priming stimulus (Ding *et al*., 2012; Lamke *et al*., 2016; D'Urso & Brickner, 2017; Liu *et al*., 2018), although different types of memory have also been described (Ding *et al*., 2013). A recent review stated that plant memory genes fit into one of two classes, showing either a sustained expression during stress recovery or hyperactivation upon re‐exposure (Bäurle & Trindade, 2020). According to the definition that memory genes show an altered response to a given repeated stimulus (Avramova, 2015; Lämke & Bäurle, 2017), the present study reports TM events that mainly lead, instead, to hypoactivation.

The decrease in cold‐stress responses suggests that *B. distachyon* habituated to cold stress under diurnal‐freezing conditions (Figs 2a, 3d,e, 6). ‘Habituation’ is a reversible response that becomes weaker over time (Rankin *et al*., 2009; Gagliano *et al*., 2018), and it has previously been used to describe plant responses to mechanical stimuli (Gagliano *et al*., 2014), cell cultures growing autonomously from hormones and growth factors (Christou, 1988; Pischke *et al*., 2006) or from toxins and growth inhibitors (Brochu *et al*., 2010; Mélida *et al*., 2010), and more recently, along with the term ‘sensitization’, to describe TMs in the context of the abiotic stress response (Liu *et al*., 2014; Liu & Avramova, 2016; Csermely *et al*., 2020). In general, the explanations of habituation responses relate to energy balance, suggesting that organisms mitigate the energy cost of constantly responding to a given stimulus (Duijn, 2017). In our system, *B. distachyon* shows reversible hypoactivation of cold‐regulated genes and can fully acclimate by resuming growth under diurnal‐freezing conditions (Figs 3d,e, 5b; Mayer *et al*., 2020). Hence, habituation likely coincided, along with the return of growth‐associated gene expression, with a diversion of the energy spent on initial stress responses towards the acquisition of a freezing‐tolerant plant morphology.

### The regulation of phenotypic plasticity

The phenotype acquired under diurnal‐freezing conditions is the result of dynamic signals that, over time, led to converging stress and growth responses. Recovery from stress is a critical period during which stress memories can either be discarded, encoded for future responses, or reinforced to maintain the expression stress‐activated genes (e.g. *VRN1*; Crisp *et al*., 2016). In Arabidopsis, recovery from cold exposure, or deacclimation, induces rapid hormonal, structural, metabolic and transcriptomic changes that relate to growth and development, and are likely to kickstart growth in spring (Zuther *et al*., 2015; Pagter *et al*., 2017). The higher temperatures of diurnal‐freezing cycles allow *B. distachyon* to grow and gain high freezing tolerance, unlike constant low temperature which can also induce chilling stress in the species (Fig. 3b; Mayer *et al*., 2020). Hence, the alternation of low/high temperature signals experienced during diurnal‐freezing conditions, or stress/recovery, appears to favour an equilibrium between stress response and growth, which could explain the ‘carried‐over’ expression of growth‐associated genes during cold exposure, and the dampening of acute cold‐stress responses over time (Figs 4e, S2–S4, S6; Table 3). Similarly, transcripts of *IRI*, *CBF*s and *VRN1* gradually accumulate at recovery time points under diurnal freezing, hence gaining higher basal expression outside of cold exposure (Fig. 3e). The gradual loss of TM of *VRN1* and the rapid loss associated with *IRI* at recovery time points also indicate that the extended expression of cold‐activated genes into the high temperatures of diurnal freezing is an acquired response to the treatment (Fig. 5a). The diurnal‐freezing phenotype therefore implies a gradual convergence of cold‐stress and growth responses. It is important to consider that, although this treatment models seasonal cues, the plasticity displayed by *B. distachyon* in response to diurnal freezing can result from both cold‐adaptive plasticity traits and conditions of the treatment itself (Fig. 4; Tables 1, 5). Although the experiments of this study do not allow for differentiation between these factors, it is probably a combination of the two. For instance, plants exposed to diurnal freezing become fully vernalized (Mayer *et al*., 2020), and the establishment of similar TMs was observed in repeated chilling and diurnal freezing conditions, although these induce contrasting responses in *B. distachyon* (Figs 2a, 3d; Mayer *et al*., 2020), which supports the idea that the shaping of cold responses via TMs likely regulates cold acclimation in *B. distachyon*.

Cold acclimation and vernalization are responses that ensure persistence in temperate climates. The expression of these two adaptive plasticity responses is linked in temperate grasses, notably through the expression of *VRN1* (Galiba *et al*., 2009; Dhillon *et al*., 2010; Deng *et al*., 2015; Mayer *et al*., 2020). This study shows that the regulatory mechanism of vernalization, namely the epigenetic regulation of *VRN1*’s TM, may also regulate cold acclimation in the temperate grass *B. distachyon* (Figs 2a, 3d,e, 5b,c). Our results show that repeated cold exposure affected the levels of global DNA methylation and of H3K27me3, H3K4me2 and H3K4me3 at the loci of genes involved in cold acclimation in *B. distachyon* (Figs 1c, 2b, 7b,c). In Arabidopsis, H3K4me3 and H3K4me2 mark hyperactive memory genes during heat stress, and mediate tolerance to salt and salt‐stress priming responses (Shen *et al*., 2014; Feng *et al*., 2016; Lamke *et al*., 2016). While H3K4me2 is usually associated with gene repression (Lämke & Bäurle, 2017; Liu *et al*., 2019), H3K4me3 is deposited on active cold‐regulated genes in potato exposed to cold conditions, along with the repressive mark H3K27me3 (Zeng *et al*., 2019). However, studies show that levels of H3K27me3 and H3K4me3 tend to be negatively correlated to one another, especially in TMs linked to vernalization in Arabidopsis, barley and *B. distachyon* – this is also supported by our results (Figs 7, S11; Finnegan *et al*., 2005; Oliver *et al*., 2009; Zhang *et al*., 2009; Huan *et al*., 2018). Our results suggest that depletions of H3 and H3K27me3 were linked to TMs established under diurnal‐freezing conditions, that H3K4me3 participated in their maintenance during recovery and the enrichment in H3K4me2 occurred in a second response to diurnal freezing (Figs 7d, S9). It is intriguing that this change in epigenetic signature occurred while the progression of transcriptional responses to diurnal freezing did not change during the second exposure (Fig. 7). Extended recovery from diurnal freezing hence induced the onset of different chromatin marks, also leading to a slight decrease in global DNA methylation upon re‐exposure, rather than a hypermethylation as observed during the first priming episode (Fig. 7c). Because cold acclimation extends to morphology in *B. distachyon*, and because vernalization occurs simultaneously, the chromatin responses observed during diurnal freezing can be related to stress memories as well as development (e.g. linked to vernalization and growth stages), demonstrating complex links between chromatin marks, and stress and development responses in *B. distachyon*.

### Conclusion

This study is the first to report that reversible TMs mediate the progressive return of plant growth following initial stress responses. By regulating transcriptional habituation, TMs provide plasticity to *B. distachyon*'s stress responses, to develop a freezing tolerant morphology during cold acclimation. Hence, chromatin‐associated TMs are involved in regulating both stress and developmental components of cold‐climate adaptive plasticity in *B. distachyon*.

## Author contributions

BFM and J‐BC designed the research, BFM performed the experiments and analyses, BFM and J‐BC wrote the manuscript.

## Supporting information


**Fig. S1** Whole‐plant freeze tests performed on plants exposed to one and four cycles of diurnal freezing, compared to non‐acclimated plants.
**Fig. S2** Significantly enriched gene ontology (GO) terms in the 17 categories regrouped into 6 expression profiles identified in diurnal‐freezing responsive genes.
**Fig. S3** Transcript levels of genes whose expression changed under diurnal‐freezing conditions (S4/S1) that are associated with significantly enriched GO terms.
**Fig. S4** Distribution and differential expression of diurnal‐freezing responsive genes in abiotic stress response modules.
**Fig. S5** Quantitative reverse transcription polymerase chain reaction (RT‐qPCR) validation of RNA‐seq analysis of plants exposed to diurnal freezing.
**Fig. S6** Families of transcription factors in the six expression profiles identified under diurnal freezing.
**Fig. S7** RT‐qPCR validation of RNA‐seq analysis of primed plants exposed to chilling.
**Fig. S8** *Brachypodium distachyon* gene modules identified as being associated with abiotic stress responses and their distribution in chilling‐responsive genes.
**Fig. S9** Chromatin marks at the loci of genes involved in cold acclimation in response to repeated priming under diurnal freezing.
**Fig. S10** Transcript levels of CBF1–3 at stress (S) and COR410/413 at recovery (R) are positively correlated.
**Fig. S11** Correlation *R* ^2^_adj_ between epigenetic marks at COR gene loci.
**Table S1** Primers used in this study.
**Table S2** Chromatin immunoprecipitation (ChIP)‐qPCR signals and statistical difference.
**Table S3** Gene ontology analysis of chilling‐responsive genes.
**Table S4** Chilling‐responsive genes common to naïve and primed responses that show transcriptional memory and their categorization as diurnal‐freezing responsive genes.

**Table S5** Annotated chilling‐responsive memory genes common to naïve and primed responses.
Please note: Wiley Blackwell are not responsible for the content or functionality of any Supporting Information supplied by the authors. Any queries (other than missing material) should be directed to the *New Phytologist* Central Office.
