# Supplementary material for: Transcriptional memories mediate the plasticity of cold stress responses to enable morphological acclimation in Brachypodium distachyon
Source: New Phytol. 2020 Oct 26;229(3):1615–34. doi: 10.1111/nph.16945 (PMC7820978; doi:10.1111/nph.16945)
Supplement: Supplementary file 1 — Fig. S1 Whole‐plant freeze tests performed on plants exposed to one and four cycles of diurnal freezing, compared to non‐acclimated plants. Fig. S2 Significantly enriched gene ontology (GO) terms in the 17 categories regrouped into 6 expression profiles identified in diurnal‐freezing responsive genes. Fig. S3 Transcript levels of genes whose expression changed under diurnal‐freezing conditions (S4/S1) that are associated with significantly enriched GO terms. Fig. S4 Distribution and differential expression of diurnal‐freezing responsive genes in abiotic stress response modules. Fig. S5 Quantitative reverse transcription polymerase chain reaction (RT‐qPCR) validation of RNA‐seq analysis of plants exposed to diurnal freezing. Fig. S6 Families of transcription factors in the six expression profiles identified under diurnal freezing. Fig. S7 RT‐qPCR validation of RNA‐seq analysis of primed plants exposed to chilling. Fig. S8 Brachypodium distachyon gene modules identified as being associated with abiotic stress responses and their distribution in chilling‐responsive genes. Fig. S9 Chromatin marks at the loci of genes involved in cold acclimation in response to repeated priming under diurnal freezing. Fig. S10 Transcript levels of CBF1–3 at stress (S) and COR410/413 at recovery (R) are positively correlated. Fig. S11 Correlation R 2 adj between epigenetic marks at COR gene loci. Table S1 Primers used in this study. Table S2 Chromatin immunoprecipitation (ChIP)‐qPCR signals and statistical difference. Table S3 Gene ontology analysis of chilling‐responsive genes. Table S4 Chilling‐responsive genes common to naïve and primed responses that show transcriptional memory and their categorization as diurnal‐freezing responsive genes. [file NPH-229-1615-s001.pdf]

New Phytologist

***New Phytologist* Supporting Information**

Article title: **Transcriptional memories mediate the plasticity of cold stress responses to enable morphological cold acclimation in *Brachypodium distachyon***

Authors: Boris F. Mayer, Jean-Benoit Charron

Article acceptance date: 04 September 2020

The following Supporting Information is available for this article:

Fig. S1: Whole-plant freeze tests performed on plants exposed to 1 and 4 cycles of diurnal-freezing compared to non-acclimated plants.

Fig. S2: Significantly enriched GO terms in the 17 categories regrouped into 6 expression profiles identified in diurnal-freezing responsive genes.

Fig. S3: Transcript levels of genes whose expression change in diurnal-freezing (S4/S1) that are associated with significantly enriched GO terms.

Fig. S4: Distribution and differential expression of diurnal-freezing responsive genes in abiotic stress response modules.

Fig. S5: RT-qPCR validation of RNA-seq analysis of plants exposed to diurnal-freezing.

Fig. S6 Families of transcription factors in the 6 expression profiles identified in diurnal-freezing.

Fig. S7: RT-qPCR validation of RNA-seq analysis of primed plants exposed to chilling

Fig. S8: *B. distachyon* gene modules identified as being associated with abiotic stress responses and their distribution in in chilling-responsive genes.

Fig. S9: Chromatin marks at the loci of genes involved in cold acclimation in response to repeated priming in diurnal-freezing.

Fig. S10: Transcript levels of CBF1-3 at stress S and COR410/413 at recovery R are positively correlated.

Fig. S11: Correlation R^2adj between epigenetic marks at COR gene loci.

Table S1: Primers used in this study**.**

Table S2: ChIP-qPCR signals and statistical difference.

Table S3: Gene ontology analysis of chilling-responsive genes.

Table S4: Chilling-responsive genes common to naïve and primed responses that show transcriptional memory and their categorization as diurnal-freezing responsive genes.

Table S5: Annotated chilling-responsive memory genes common to naïve and primed responses.

(submitted as an excel file)

Fig. S1: Whole-plant freeze tests performed on plants exposed to 1 and 4 cycles of diurnal-freezing compared to non-acclimated plants.

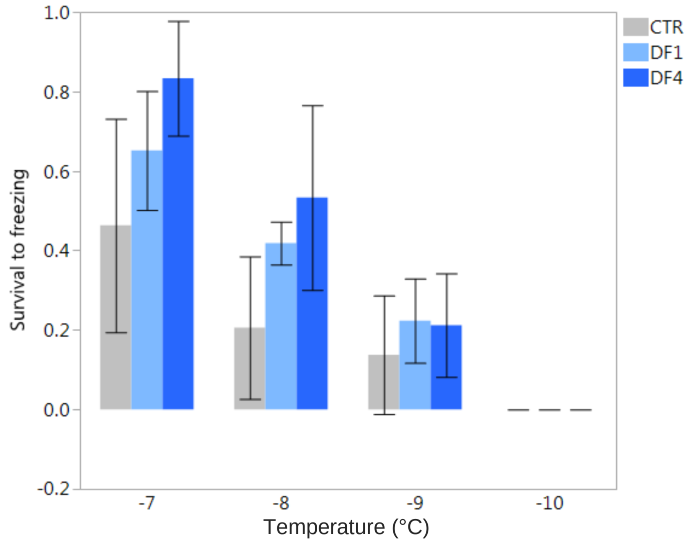

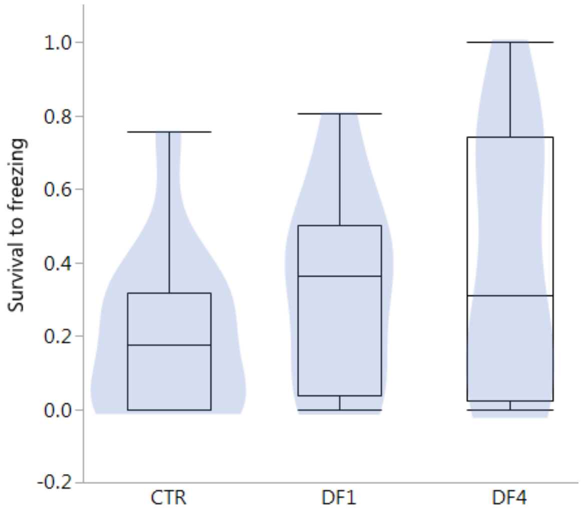

Error bars represent standard deviations between three independent survival tests.

Fig. S2: Significantly enriched GO terms in the 17 categories regrouped into 6 expression profiles identified in diurnal-freezing responsive genes.

(a) U2A, L (L1-4), U1, 2B, D2B, T(T1-4) are part of the 17 gene categories described in Table 1.
(b) C (complex-convergent and complex-divergent), L (late-responsive), S (stable) and T (transient) are part of the 6 transcriptional profiles in DFRG. NB categories that are not represented returned no significantly enriched terms.

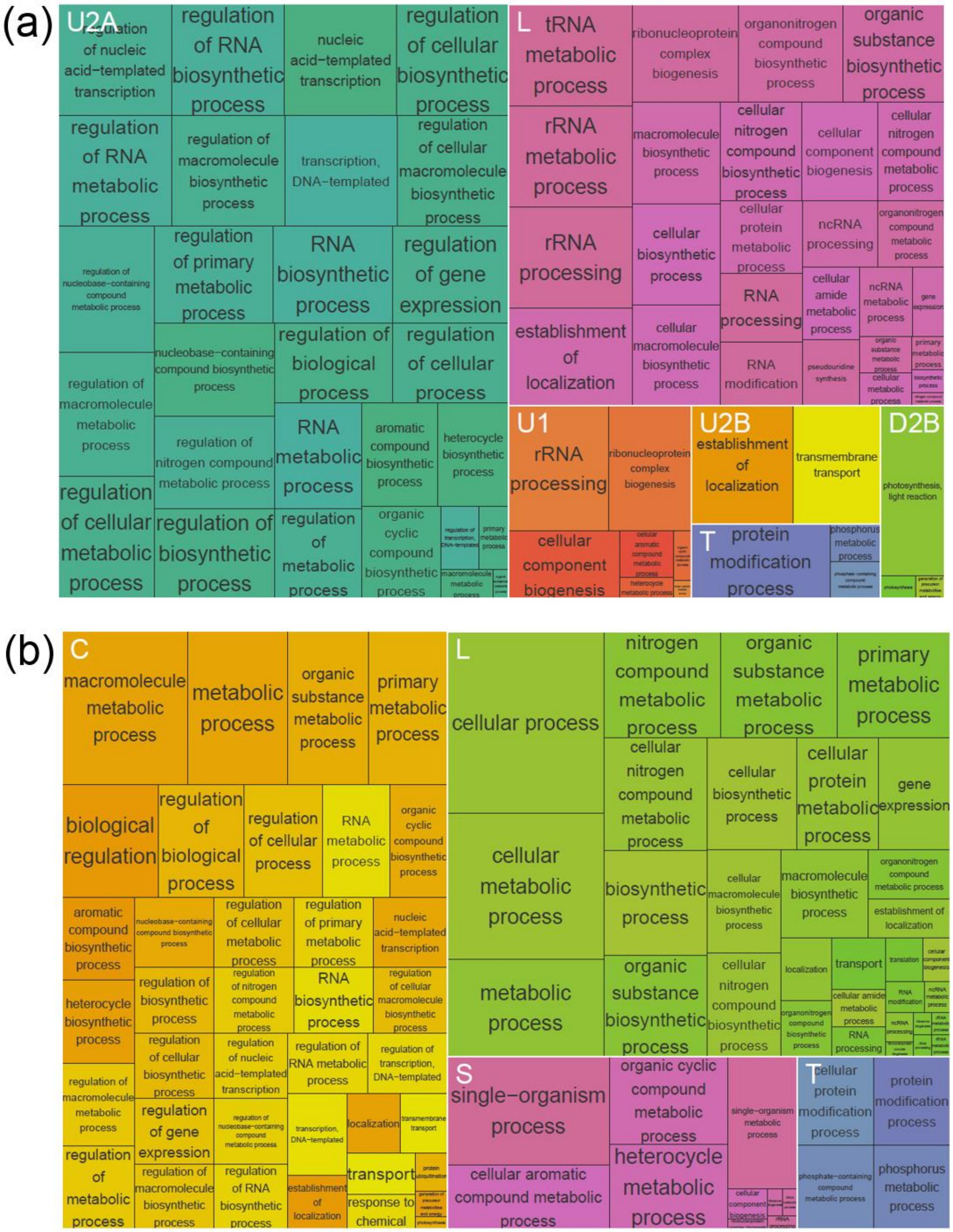

New Phytologist

Fig. S3: Transcript levels of genes whose expression change in diurnal-freezing (S4/S1) that are associated with significantly enriched GO terms.

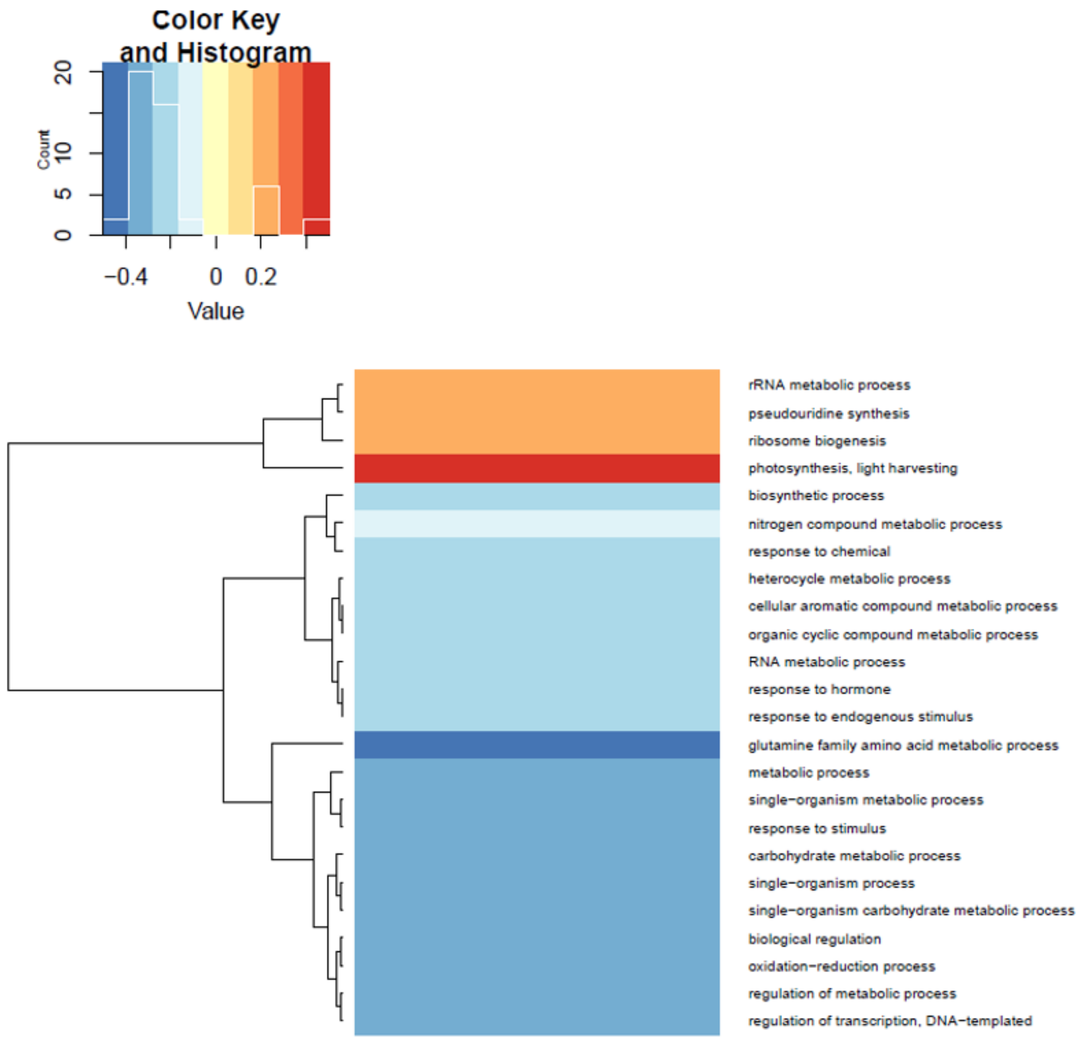

Fig. S4: Distribution and differential expression of diurnal-freezing responsive genes in abiotic stress response modules.

(a) Percent of 17 diurnal-freezing responsive gene categories which belong to previously identified modules present (in yellow to green) and total fold-change associated with each module in S1 and S4 responses (in blue to red). (b) Fold change associated with each DFRG category x gene module. Modules were identified in Priest et al., 2014 https://doi.org/10.1371/journal.pone.0087499.

(a)

% genes in category

	Up			Down			Transient				Late				Offset	Oscillating		
	Stable	higher	lower	Stable	lower	higher	Up		Down		Up		Down		U/D	d/u	u/d	Total
	U1	U2B	U2A	D1	D2A	D2B	T1	T3	T2	T4	L1	L3	L2	L4	O2	O1A	O1B	
M18	2.9		0.3	1.0		0.6	1.4	0.3	4.8	1.6		5.4		1.9			1.6	2.0
M21	0.3		0.3	0.2	1.1		0.9	1.3	0.2				4.0	0.6	3.3		4.9	1.6
M13	1.3		2.0	2.6			1.1	0.3	3.7	0.8		0.9	1.1	1.9				1.6
M16	1.4	1.4	1.0					0.3			1.4							1.1
M17	0.1								0.2		1.4							0.6
M9	0.6	2.8	0.3	2.0	1.1	1.2	0.9	0.6	2.8	2.4	1.4	2.8	0.6	1.6		6.3		1.8
M5	0.3	2.8	0.3	0.6	3.2		0.5	0.3	0.2		1.4	0.2	2.3	1.9	3.3			1.3
M10	1.9	1.4	1.3	0.4	1.6		1.4	4.1	0.2		1.4	0.2	1.7				1.6	1.4
M19	0.1		1.5	0.1			0.2	1.9	0.2		1.4	0.2	1.7					0.8
M22	0.8		0.5					0.3			1.4	0.2						0.6
**M1**	3.9	4.2	0.8	2.1	0.5	2.5	5.6	0.3	5.4	10.5	10.1	5.9	1.1	3.2		6.3	1.6	4.0
M14	0.6	4.2	1.8	0.1	0.5		0.2	2.2	0.2					0.6			1.6	1.2
M11	0.1			0.9	2.1								2.3	1.3			3.3	1.7
M3	0.8		6.6	0.2	1.6		2.5	6.7	0.4		1.4		2.9	0.3	6.7		6.6	3.1
M8	0.2		0.5	0.2			0.2		0.4			0.2		0.6		6.3		1.1
**M4**	5.5	1.4	9.2	1.5	2.1		4.3	6.7	3.5	1.6	1.4	14.1	2.3	1.3	6.7	6.3	1.6	4.3
M6	0.4			1.3		4.9	0.7		1.7	3.2		2.8		1.0				2.0
**M2**	11.9		1.0	0.6	1.1		2.9	0.6	1.5	1.6		14.8		0.3		6.3	1.6	3.7
M12	0.2		0.3	0.2	2.6		0.2						1.1	0.6	3.3	6.3		1.7
M15	0.2							0.3	0.4									0.3
M7	0.6		0.5	0.5	0.5	0.6	0.7	0.6		1.6		0.7	2.3	1.3			3.3	1.1
M20				0.4					0.4									0.4
**Other**	66	82	72	85	82	90	76	73	74	77	77	52	76	81	77	63	72	74.9

	log2(FC)		
	S1	S4	S4-S1
**M18**	2.66	2.574	-0.09
**M21**	2.40	2.0469	-0.35
M13	0.91	0.1143	-0.80
M16	0.88	0.4619	-0.41
M17	0.76	0.3167	-0.45
M9	0.72	0.4831	-0.24
M5	0.69	0.2134	-0.48
**M10**	0.66	-0.5266	-1.18
M19	0.56	-0.3843	-0.95
**M22**	0.53	1.2692	0.74
M1	0.50	0.0406	-0.46
**M14**	0.46	-0.6401	-1.10
M11	0.42	-0.0686	-0.49
M3	0.40	0.2359	-0.16
M8	0.24	0.1953	-0.04
M4	0.11	-0.0652	-0.18
M6	0.06	0.0447	-0.02
M2	0.05	0.0472	0.00
M12	-0.02	-0.6112	-0.59
**M15**	-0.26	-1.51	-1.25
M7	-0.74	-0.3801	0.36
**M20**	-1.26	-1.2083	0.05

(b)

		Up			Down			Transient				Late				Offset	Oscillating		Total
		Stable	higher	lower	Stable	lower	higher	Up		Down		Up		Down		U/D	d/u	u/d	
		U1	U2B	U2A	D1	D2A	D2B	T1	T3	T2	T4	L1	L3	L2	L4	O2	O1A	O1B	
log2(S1/S0)	M18	2.94	3.95	4.13					1.87			0.42							2.66
	M21	4.04		4.34					2.01			0.70	0.89						2.40
	M13	2.30	2.22	4.83	-1.89	-1.62		1.08	2.98	-1.03					-0.57			0.82	0.91
	M16	1.81							2.10	-1.28									0.88
	M17	2.04		3.85	-2.16			1.08	1.68	-1.16		0.88	0.72	-0.05					0.76
	M9	2.39	2.86	4.67	-1.92	-1.35		1.31	1.69	-1.83		-0.25	0.91	-0.18				0.38	0.72
	M5	2.10		4.78	-1.74			1.31	3.18	-1.21	-1.38		0.73	-0.10	-0.73				0.69
	M10	2.46		5.09	-2.30	-1.77		1.32	2.20	-1.27		0.66		-0.05	-0.60	1.38		0.75	0.66
	M19	2.09		5.13	-1.81	-2.02		1.25						0.42	-0.64	1.08	-0.42		0.56
	M22	2.66								-1.14		0.08							0.53
	M1	1.81	1.40	4.56	-1.51	-1.40		1.33	2.31	-1.31	-1.62	0.63	0.76	-0.30	-0.78	2.34	-0.96	0.79	0.50
	M14	3.19		2.81	-1.31	-2.41		1.33	1.54	-1.61				-0.10	-0.63	1.67		0.56	0.46
	M11	2.14	1.84	3.04	-1.39	-2.90		1.16	1.75	-1.28		-0.17	0.45	0.17	-0.72	1.45			0.42
	M3	1.53		5.14	-1.58		-2.10	1.12	2.48	-1.22	-1.52		0.74		-0.80			0.59	0.40
	M8	1.60		2.83	-1.34			1.08		-1.16			0.57		-0.86		-0.80		0.24
	M4	1.51		3.63	-1.64	-2.16		1.23	1.17	-1.20	-1.57		0.81		-0.33		-0.32	0.26	0.11
	M6	1.67	2.03	4.12	-1.84	-1.81	-3.51	1.17	2.12	-1.24	-1.40	0.39	0.80	0.10	-0.82		-0.82		0.06
	M2	1.73	2.84	4.30	-1.78	-2.82	-2.64	1.19	1.94	-1.27	-1.88	0.24	0.72	-0.53	-0.84		-0.66	0.26	0.05
	M12	3.66		2.81	-2.05	-3.81	-2.16	1.32	1.86		-2.09		0.78	-0.23	-0.86			0.50	-0.02
	M15	5.01			-2.62	-3.83								-0.52	-0.35			0.72	-0.26
	M7	1.36			-2.10		-3.23	1.30		-1.35	-1.83		0.70		-0.78				-0.74
	M20				-1.46					-1.06									-1.26
log2(S4/S0)	M18	2.71	5.25	2.58					0.28			2.05							2.574
	M21	3.88		2.66					0.83			1.83	1.03						2.047
	M13	2.19	3.76	2.86	-1.43	-4.47		0.35	0.28	-0.85					-1.08			-0.44	0.114
	M16	1.32							0.95	-0.88									0.462
	M17	1.20		2.25	-2.49			0.85	-0.04	-0.64		2.06	1.59	-1.92					0.317
	M9	2.29	4.79	2.97	-2.08	-2.59		0.65	0.31	-0.84		1.76	1.21	-1.96				-0.72	0.483
	M5	2.00		2.71	-1.58			0.67	1.00	-0.76	-0.32		1.27	-1.48	-1.37				0.213
	M10	2.19		2.78	-3.09	-4.49		0.65	0.27	-0.90		1.74		-1.92	-1.39	-1.66		-0.49	-0.527
	M19	1.61		3.73	-1.94	-3.72		0.69						-1.28	-1.34	-1.94	0.73		-0.384
	M22	3.05								-0.91		1.67							1.269
	M1	1.77	2.83	2.56	-1.46	-3.16		0.81	0.38	-0.76	-0.56	1.72	1.19	-1.61	-1.14	-1.59	0.14	-0.46	0.041
	M14	2.99		1.67	-1.72	-3.66		0.61	0.02	-0.97				-1.75	-1.44	-2.18		-0.60	-0.64
	M11	2.03	3.80	1.89	-1.57	-5.01		0.85	0.37	-0.78		1.39	1.39	-1.92	-1.25	-2.09			-0.069
	M3	1.61		3.52	-1.46		-1.08	0.95	0.98	-0.72	-0.24		1.15		-1.21			-0.90	0.236
	M8	1.26		1.45	-1.58			0.75		-0.74			1.09		-1.05		0.38		0.195
	M4	1.65		1.95	-1.56	-3.33		0.89	-0.13	-0.39	0.13		1.23		-1.30		0.89	-0.82	-0.065
	M6	1.77	3.88	1.97	-1.68	-3.49	-1.78	0.69	0.60	-0.71	-0.30	1.63	1.26	-2.17	-1.37		0.39		0.045
	M2	1.77	4.35	2.54	-1.70	-5.45	-1.26	0.70	0.84	-0.69	-0.28	2.10	1.21	-1.63	-1.32		0.40	-0.83	0.047
	M12	3.40		1.68	-2.09	-6.29	-1.08	0.72	0.31		-0.06		1.27	-3.20	-1.30			-0.69	-0.611
	M15	4.84			-2.99	-6.81								-2.36	-1.18			-0.55	-1.51
	M7	1.53			-1.82		-1.89	0.67		-0.84	-0.67		1.17		-1.19				-0.38
	M20				-1.53					-0.88									-1.208

Notes:

The modules previously identified in *B. distachyon* characterize the plasticity of abiotic stress responses, and as such allow to determine if the diurnal-freezing treatment induces specific responses or changes during cold acclimation. Around 25% of diurnal-freezing responsive genes (1702 genes) belonged to one of the 22 modules identified in Priest *et al.* (2014). Modules 18 and 21, previously identified as cold-responsive, were the most differentially expressed in S1 and S4, while the expression of modules 7, 10, 14, 15 and 22 changed the most between S1 and S4, and the highest number of diurnal-freezing responsive genes were found in modules 1, 2 and 4. Although modules 14, 15 and 22 are not significantly enriched in specific GO, module 7 – upregulated in S4/S1 – is enriched in photosynthesis genes, and module 10 – downregulated in S4/S1 – is enriched in transcription factors. Module 1 and 4 are associated with gene expression, and 2 with the regulation of chromatin structure, replication and growth. As module 2 included mostly *late-responsive* genes expressed in the S4 response, S4 is associated with restored photosynthesis and growth-related responses, and S1 with transiently expressed transcription factors.

Fig. S5: RT-qPCR validation of RNA-seq analysis of plants exposed to diurnal-freezing.

Genes identified in the RNA-seq analysis in response to one (S1) or 4 (S4) cycles of diurnal-freezing that were responsive but showed no memory (a), whose transcript levels changed over time in diurnal-freezing (b) or that were not found to be responsive to the treatment (fold change lower than 2; c). This analysis was performed on three biological replicates from experiments replicated in time, error bars show standard deviation between these.

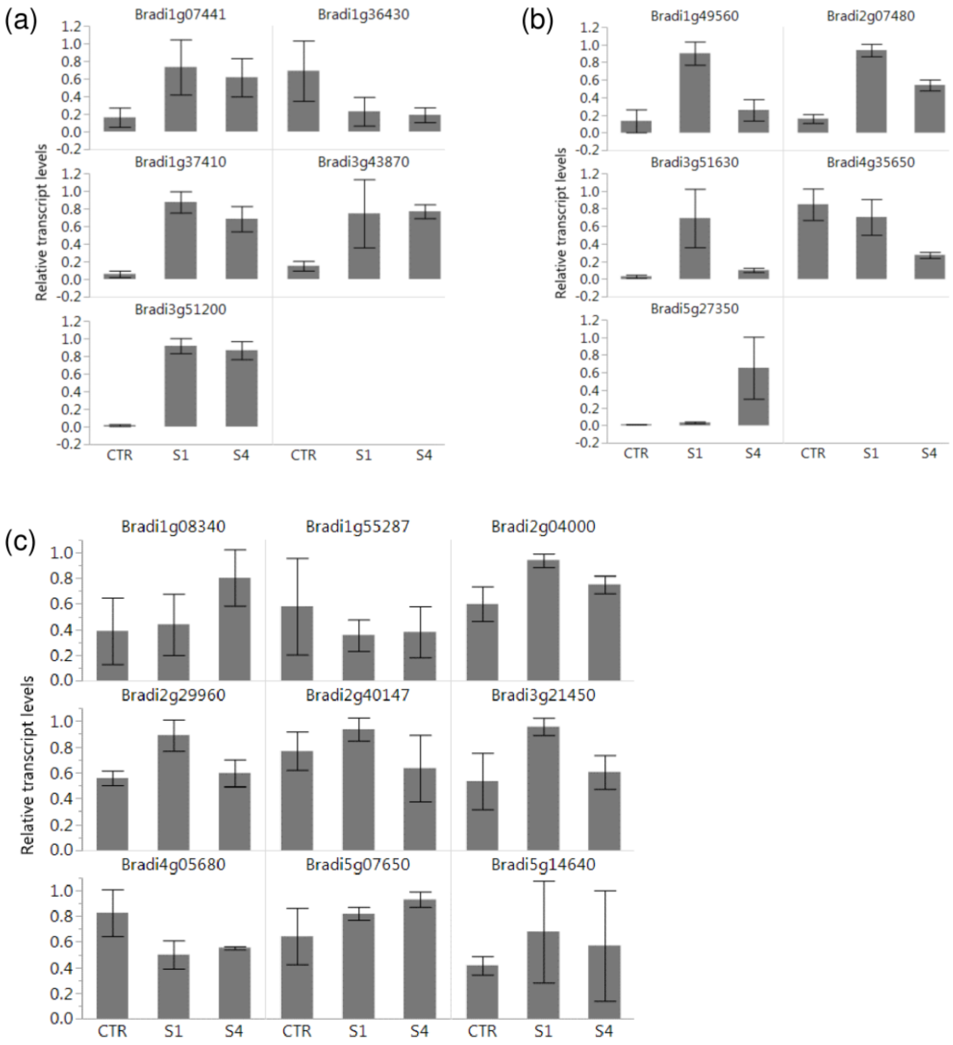

Fig. S6: Families of transcription factors in the 6 expression profiles identified in diurnal-

freezing.

(a) Distribution of transcription factor and transcriptional regulator families in stable (S), complex-convergent (CC), complex-divergent (CD), transient (T), late-responsive (L) and offset/oscillating (O) genes. (b) Distribution of transcription factor and transcriptional regulator families in CD, CC, T, L and O, excluding the families specific to S. (c) Distribution of transcription factor and transcriptional regulator families in memory (CCo, CD and strong T) and non-memory genes (S, L, O and weak T).

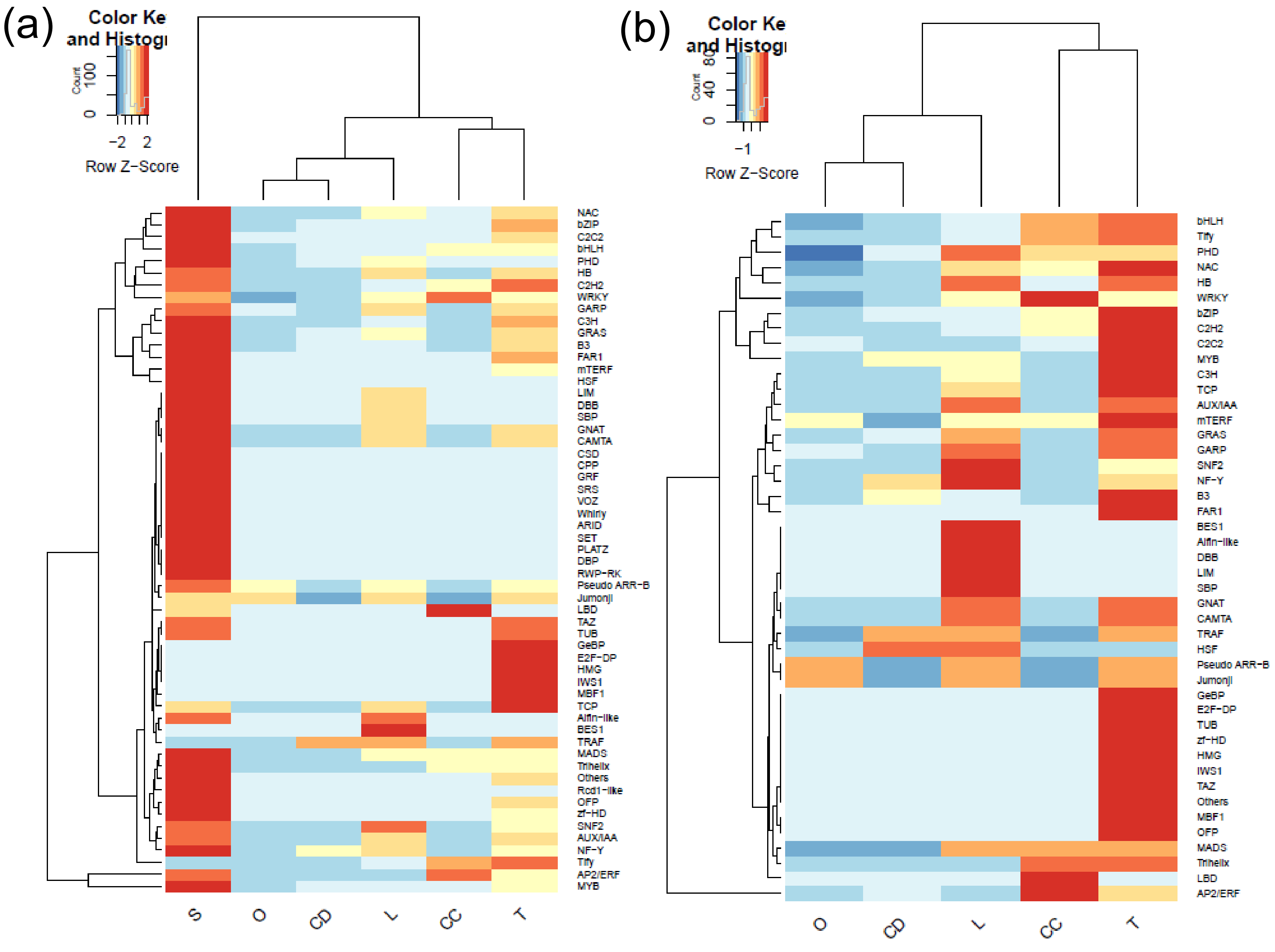

(c)

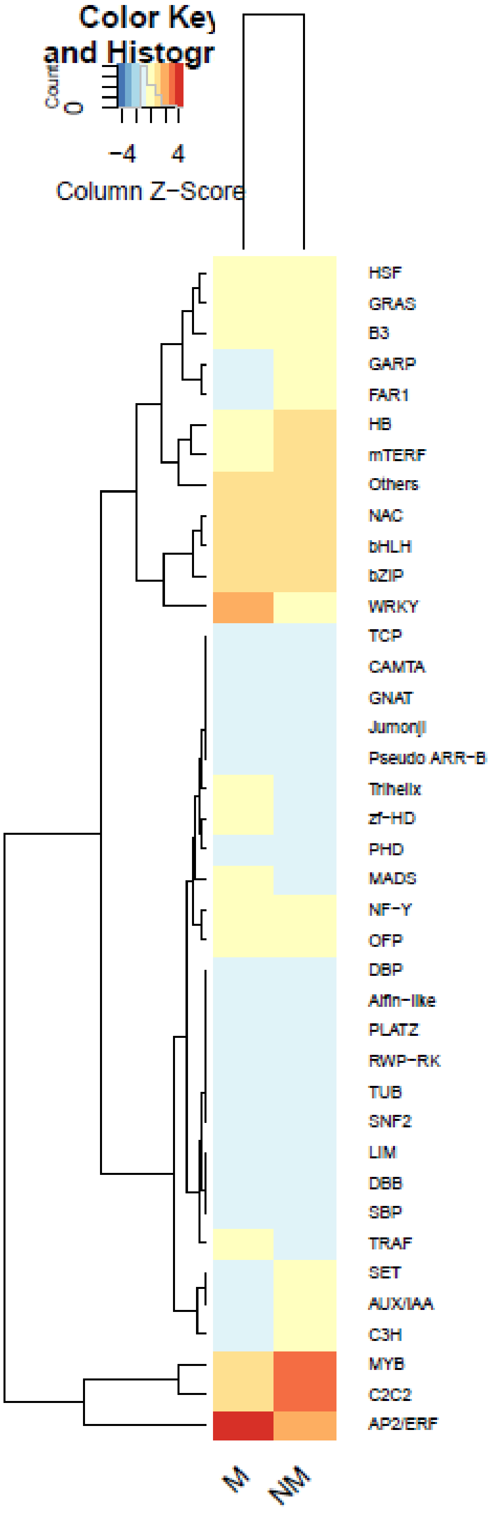
Color Key and Histogr
Count
0
−4
4
Column Z−Score
HSF
GRAS
B3
GARP
FAR1
HB
mTERF
Others
NAC
bHLH
bZIP
WRKY
TCP
CAMTA
GNAT
Jumonji
Pseudo ARR−B
Trihelix
zf−HD
PHD
MADS
NF−Y
OFP
DBP
Alfin−like
PLATZ
RWP−RK
TUB
SNF2
LIM
DBB
SBP
TRAF
SET
AUX/IAA
C3H
MYB
C2C2
AP2/ERF
M
NM

Fig. S7: RT-qPCR validation of RNA-seq analysis of primed plants exposed to chilling.

Genes identified in the RNA-seq analysis in plants primed in diurnal-freezing and exposed to chilling (P) compared to naïve plants (N) and non-stressed primed plants (CTRP) and non-stressed control plants (CTR) that showed, according to the RNA-seq analysis, the establishment of memory in response to diurnal-freezing (a), or showed no change in response to chilling after priming in diurnal-freezing (no memory; b), or that showed no response to chilling (fold change lower than 2; c). This analysis was performed on three biological replicates from experiments replicated in time, error bars show standard deviation between these.

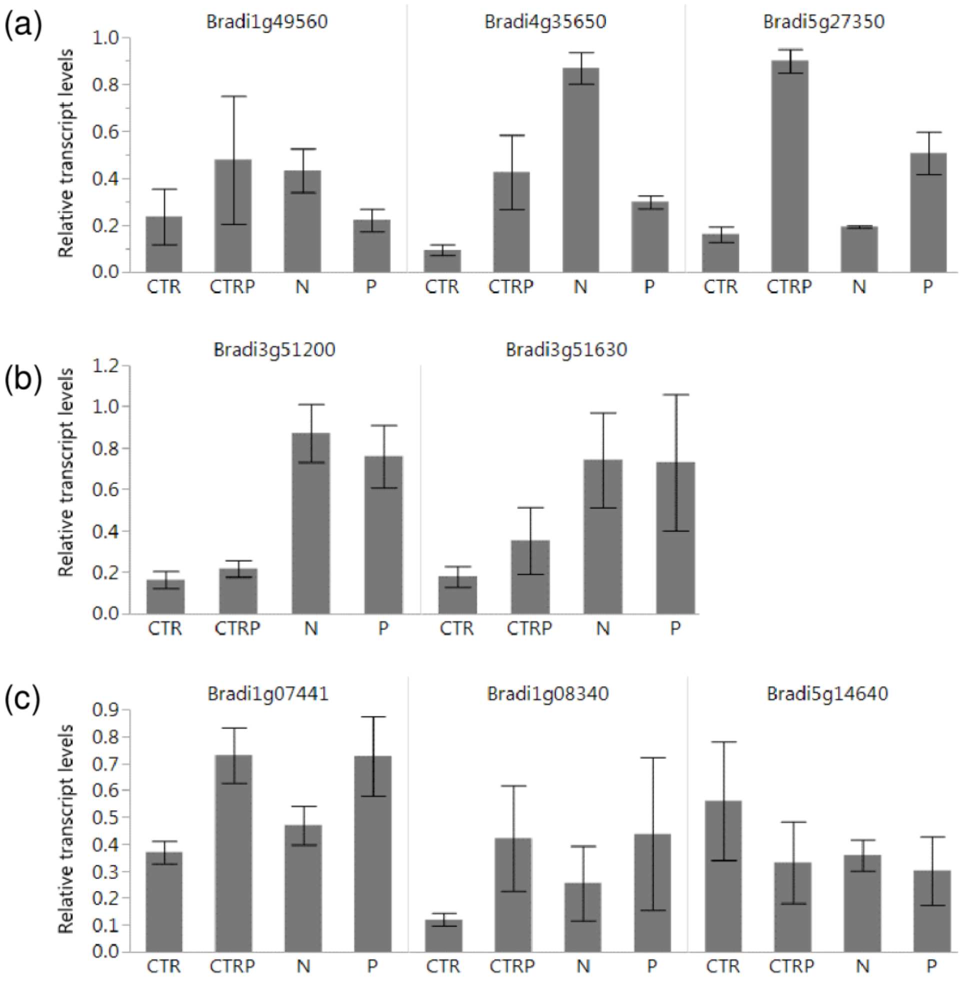

Fig. S8: B. distachyon gene modules identified in abiotic stress response and their distribution in in chilling-responsive genes

by identity to modules

	uniN	uniP	shared
1	51	7	14
2	18	8	41
3	7	0	0
4	1	4	4
5	11	1	4
6	4	0	2
7	6	1	3
8	0	0	0
9	7	3	2
10	2	7	11
11	2	0	0
12	16	25	16
14	2	2	2
15	0	1	0
16	6	13	1
17	12	2	7
18	12	2	14
19	14	1	3
20	8	14	0
21	0	0	1
22	11	8	2

NB: *Number of genes unique to the naïve response (uniN), to the primed response (uniP) or that are found in both naïve and primed responses (shared). Modules were identified in Priest et al., 2014 https://doi.org/10.1371/journal.pone.*0087499. *Stress-response transcription factors AP2/ERF, bHLH, WRKY and C2C2 are found in module 18. Colors highlight the number of genes found in each module from loe to high (blue to red).*

Fig. S9: Chromatin marks at the loci of genes involved in cold acclimation in response to repeated priming in diurnal-freezing

Levels of histone H3 relative to input DNA (top left), H3K27me3 (top right), H3K4me2 (bottom left) and H3K4me3 (bottom right) relative to H3 on promoters of *CBFs*, *IRI*, *COR410-413* and *VRN1* and within the first intron of *VRN1* (VRN1_R1) in CTR, P1, R and P2.

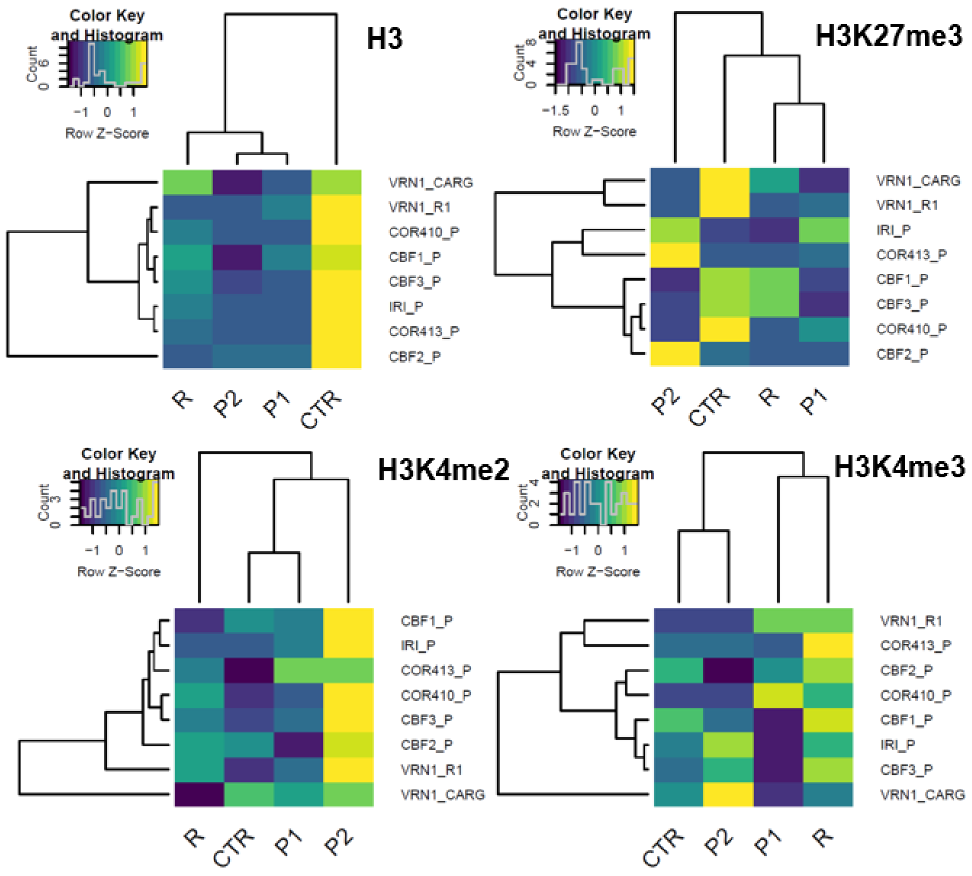

Fig. S10: Transcript levels of CBF1-3 at stress S and COR410/413 at recovery R are positively correlated.

(a) Relative transcript levels of averaged CBF1-3 sampled at the stress time-point in diurnal-freezing (CBF (S)) and COR410-COR413 sampled at the recovery time-point (COR (R)) in naïve (N, N2, DF1), primed (P, DF4), primed in lag for 1, 3, 6 and 9 days and triggered by 1 cycle of DF (L1-9T). NB at all time points, CBF (S) and COR (R) were not found to be statistically different. *Error bars represent standard deviation between three biological replicates.* (b) Linear regression between CBF (S) and COR (R). COR = 0.019517 + 1.0116828*CBF; R^2_{adj}=0.812; Prob > F = 0.0014.

(a)

(b)

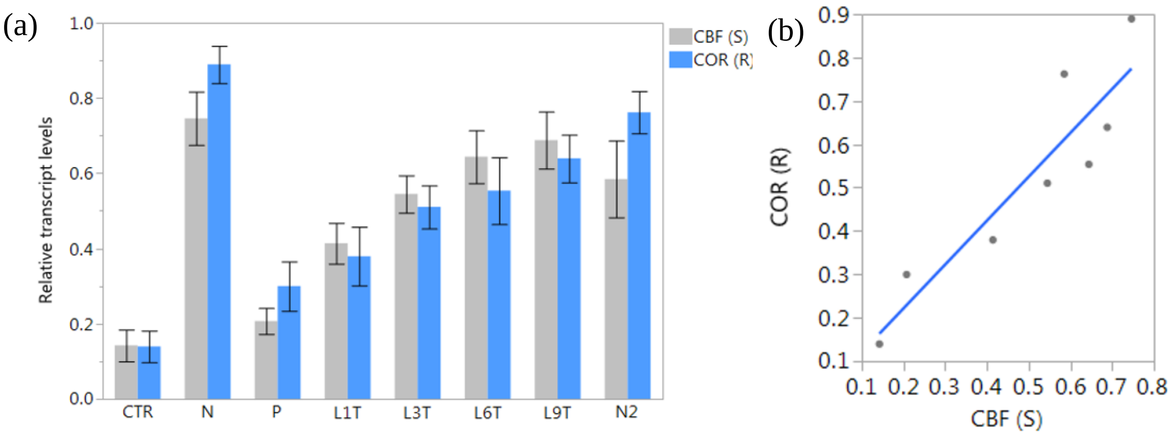

Fig. S11: Correlation R^2adj between epigenetic marks at COR gene loci

Multivariate target=CBF1_P

Correlations

	ip.h3	ip.k27	ip.k4me2	ip.k4me3
ip.h3	1.0000	0.8130	-0.5908	0.5192
ip.k27	0.8130	1.0000	-0.6295	0.9198
ip.k4me2	-0.5908	-0.6295	1.0000	-0.5221
ip.k4me3	0.5192	0.9198	-0.5221	1.0000

Multivariate target=COR413_P

Correlations

	ip.h3	ip.k27	ip.k4me2	ip.k4me3
ip.h3	1.0000	-0.3387	-0.9246	-0.2078
ip.k27	-0.3387	1.0000	0.4869	-0.2940
ip.k4me2	-0.9246	0.4869	1.0000	-0.1787
ip.k4me3	-0.2078	-0.2940	-0.1787	1.0000

Multivariate target=CBF2_P

Correlations

	ip.h3	ip.k27	ip.k4me2	ip.k4me3
ip.h3	1.0000	-0.1950	-0.0358	0.1843
ip.k27	-0.1950	1.0000	0.7772	-0.9365
ip.k4me2	-0.0358	0.7772	1.0000	-0.5108
ip.k4me3	0.1843	-0.9365	-0.5108	1.0000

Multivariate target=IRI_P

Correlations

	ip.h3	ip.k27	ip.k4me2	ip.k4me3
ip.h3	1.0000	-0.6501	-0.4670	-0.0446
ip.k27	-0.6501	1.0000	0.7573	-0.0287
ip.k4me2	-0.4670	0.7573	1.0000	0.6252
ip.k4me3	-0.0446	-0.0287	0.6252	1.0000

Multivariate target=CBF3_P

Correlations

	ip.h3	ip.k27	ip.k4me2	ip.k4me3
ip.h3	1.0000	0.8018	-0.7379	-0.1059
ip.k27	0.8018	1.0000	-0.4899	0.4658
ip.k4me2	-0.7379	-0.4899	1.0000	0.4790
ip.k4me3	-0.1059	0.4658	0.4790	1.0000

Multivariate target=VRN1_CARG

Correlations

	ip.h3	ip.k27	ip.k4me2	ip.k4me3
ip.h3	1.0000	0.8563	-0.4901	-0.3979
ip.k27	0.8563	1.0000	-0.0416	0.0141
ip.k4me2	-0.4901	-0.0416	1.0000	0.4107
ip.k4me3	-0.3979	0.0141	0.4107	1.0000

Multivariate target=COR410_P

Correlations

	ip.h3	ip.k27	ip.k4me2	ip.k4me3
ip.h3	1.0000	0.9172	-0.6832	-0.4602
ip.k27	0.9172	1.0000	-0.8349	-0.2392
ip.k4me2	-0.6832	-0.8349	1.0000	-0.3131
ip.k4me3	-0.4602	-0.2392	-0.3131	1.0000

Multivariate target=VRN1_R1

Correlations

	ip.h3	ip.k27	ip.k4me2	ip.k4me3
ip.h3	1.0000	-0.4237	-0.7762	-0.4940
ip.k27	-0.4237	1.0000	0.8723	-0.5673
ip.k4me2	-0.7762	0.8723	1.0000	-0.1590
ip.k4me3	-0.4940	-0.5673	-0.1590	1.0000

NB: Colours indicate the value of R^2adj from red to grey to blue, where negative R^2adj are red and positive R^2adj are blue. Highest correlations between chromatin marks were highlighted in yellow.

Table S1: Primers used in this study

Primer	Analysis	Primer sequence	Reference
UBC18_F	RT-qCPR	GTCACCCGCAATGTCTGTAAGTTC	Ream et al. 2014
UBC18_R	RT-qCPR	TTGTCTTGCGGACGTTGCTTTG	
VRN1_F	RT-qCPR	GCTCTGCAGAAGGAACTTGTGG	Ream et al. 2014
VRN1_R	RT-qCPR	CTAGTTTGCGGGTGTGTTTGCTC	
CBF1_F	RT-qCPR	ACCCGTACTACGAGATGGGC	Ryu et al. 2014
CBF1_R	RT-qCPR	ATCGGAGGAGGGTCAATGAG	
CBF2_F	RT-qCPR	GTGGCGCAGTCGTCTTCTT	Ryu et al. 2014
CBF2_R	RT-qCPR	GCTGGTCCTGCPAGTCACAC	
CBF3_F	RT-qCPR	TCGTCCTCCCTCACTGACAA	Ryu et al. 2014
CBF3_R	RT-qCPR	GCGTAGTAGAGGTCCCAGCC	
IR_F	RT-qCPR	TCTGGGACCTACCATGTCGT	Mayer et al. 2020
IRI_R	RT-qCPR	CGGACATGAGCTTCGTCAGT	
COR410_F	RT-qCPR	AGCAAAAGCCACAAGCCAAG	Mayer et al. 2020
COR410_R	RT-qCPR	GTCAAAGAGGCCCCTATCCG	
COR413_F	RT-qCPR	AGGTTGGTTGCTGGATTGCGTTC	Colton-Gagnon et al. 2014
COR413_R	RT-qCPR	TCCAGCCAATCAGGAAAGTGGCG	
VRN1_CArG_F	ChIP-qPCR	CGACAACGGATATGCTCCAGACC	Woods et al. 2017
VRN1_CArG_R	ChIP-qPCR	GAAGAGAGCCGGAGAGTGGGT	
VRN1_I1_F	ChIP-qPCR	TACGCACGCCTACGCTTAAG	Woods et al. 2017
VRN1_I1_R	ChIP-qPCR	GAAATGGAGCAGACAGGCAAG	
CBF1_R1_F	ChIP-qPCR	CAAGAGCAGAGTAGCCCAGC	Mayer et al. 2020
CBF1_R1_R	ChIP-qPCR	GGCGTTAACTGGGTCGGAAC	
CBF2_R1_F	ChIP-qPCR	TTTGGCGGGATCTCTTGCAT	Mayer et al. 2020
CBF2_R1_R	ChIP-qPCR	CGGGATTGCTATGCGTGTTG	
CBF3_R1_F	ChIP-qPCR	CGGTTGTACGGTATGTCGCT	Mayer et al. 2020
CBF3_R1_R	ChIP-qPCR	AAATCTCCGCTGGAGGAACC	
IR_R1_F	ChIP-qPCR	TGCCCACTCCATACAACACC	This paper
IRI_R1_R	ChIP-qPCR	TGCAAAGTTAGTAGCGAAGGAGT	
COR410_R1_F	ChIP-qPCR	TGGAGGTAACGGATAGGGGC	This paper
COR410_R1_R	ChIP-qPCR	TTCACCGTCACGAGGTTAGTA	
COR413_R1_F	ChIP-qPCR	GCATCCTGAAGGCTGAATCC	This paper
COR413_R1_R	ChIP-qPCR	AATTCCGGGGGTAAACGTCG	

Colton-Gagnon K, Ali-Benali MA, Mayer BF, Dionne R, Bertrand A, Do Carmo S, Charron JB. 2014. Comparative analysis of the cold acclimation and freezing tolerance capacities of seven diploid Brachypodium distachyon accessions. *Annals of Botany* **113**(4): 681-693.

Mayer BF, Bertrand A, Charron J-B. 2020. Treatment Analogous to Seasonal Change Demonstrates the Integration of Cold Responses in *Brachypodium distachyon*. *Plant Physiology* **182**(2): 1022-1038.

Ream TS, Woods DP, Schwartz CJ, Sanabria CP, Mahoy JA, Walters EM, Kaeppler HF, Amasino RM. 2014. Interaction of photoperiod and vernalization determines flowering time of Brachypodium distachyon. *Plant Physiology* **164**(2): 694-709.

Ryu JY, Hong SY, Jo SH, Woo JC, Lee S, Park CM. 2014. Molecular and functional characterization of cold-responsive C-repeat binding factors from Brachypodium distachyon. *BMC Plant Biology* **14**(1): 15.

Woods DP, Ream TS, Bouché F, Lee J, Thrower N, Wilkerson C, Amasino RM. 2017. Establishment of a vernalization requirement in *Brachypodium distachyon* requires *REPRESSOR OF VERNALIZATION1*. *Proceedings of the National Academy of Sciences* **114**(25): 6623-6628.

Table S2: ChIP-qPCR signals and statistical difference

(a) Results presented in a heatmap in Fig. 1d. (b) Results presented in a heatmap in Fig. 2b. SD: standard deviation; different letters refer to statistically significant differences between treatments. Signals relative to H3, except for H3 relative to input.

(a)

		CTR-14		C1-14		R-3		C2-7	
		Mean	SD	Mean	SD	Mean	SD	Mean	SD
CBF1	H3	2.257	± 0.07 [b]	0.93	± 0.34 [c]	1.119	± 0.29 [c]	4.02	± 0.32 [a]
	H3K27me3	6.135	± 1.28 [ab]	8.292	± 1.46 [a]	4.881	± 1.52 [b]	1.193	± 0.15 [c]
	Mock	0.874	± 0.27	0.337	± 0.58	0.823	± 0.16	0.296	± 0.05
COR410	H3	9.549	± 1.25 [a]	1.64	± 0.29 [c]	4.997	± 0.16 [b]	6.098	± 0.3 [b]
	H3K27me3	7.726	± 0.56 [a]	5.866	± 1.29 [a]	3.575	± 0.45 [b]	1.137	± 0.26 [c]
	Mock	0.379	± 0.11	0.694	± 0.19	1.582	± 0.08	0.142	± 0.1
IRI	H3	11.24	± 0.86 [a]	2.933	± 0.47 [b]	9.467	± 0.67 [c]	19.13	± 0.56 [d]
	H3K27me3	10.9	± 1.22 [a]	4.383	± 1.45 [b]	4.892	± 0.4 [b]	1.348	± 0.05 [c]
	Mock	0.281	± 0.08	0.637	± 0.09	0.825	± 0.18	0.036	± 0.02
VRN1	H3	10.21	± 0.6 [a]	2.534	± 0.34 [b]	5.762	± 1.23 [c]	20.09	± 1.05 [d]
	H3K27me3	29.87	± 3.23 [a]	11.59	± 1.26 [c]	19.55	± 1.52 [b]	12.76	± 0.27 [c]
	Mock	0.333	± 0.01	2.612	± 0.64	1.724	± 0.05	0.324	± 0.11

(b)

		CTR (3h)		C1 (3h)		R (3h)		C2 (3h)	
		Mean	SD	Mean	SD	Mean	SD	Mean	SD
CBF1	H3	5.62	± 0.16 [a]	3.20	± 0.35 [c]	4.49	± 0.54 [b]	2.33	± 0.29 [c]
	H3K27me3	1.75	± 0.73 [a]	1.82	± 0.33 [a]	2.63	± 0.4 [a]	1.71	± 0.36 [a]
	H3K4me2	0.95	± 0.15 [ab]	0.57	± 0.13 [b]	1.38	± 0.4 [a]	1.05	± 0.28 [ab]
	H3K4me3	13.08	± 0.9 [c]	17.89	± 0.51 [bc]	24.33	± 2.18 [a]	19.23	± 2.8 [b]
	Mock	0.67	± 0.2	0.35	± 0.49	0.18	± 0.08	0.17	± 0.16
COR410	H3	11.92	± 1.26 [a]	11.20	± 1.15 [a]	7.05	± 1.09 [b]	6.78	± 0.7 [b]
	H3K27me3	1.14	± 0.39 [a]	1.10	± 0.29 [a]	0.63	± 0.08 [ab]	0.36	± 0.09 [b]
	H3K4me2	1.39	± 0.18 [ab]	0.64	± 0.35 [b]	1.76	± 0.31 [a]	0.79	± 0.37 [b]
	H3K4me3	20.44	± 3.79 [b]	14.49	± 2.45 [b]	57.13	± 7.58 [a]	12.70	± 0.89 [b]
	Mock	0.67	± 0.3	0.30	± 0.07	0.29	± 0.33	0.26	± 0.17
IRI	H3	12.49	± 0.9 [a]	12.63	± 1.58 [a]	10.65	± 1.16 [ab]	8.84	± 1.07 [b]
	H3K27me3	5.28	± 1.11 [a]	5.94	± 0.86 [a]	2.61	± 0.63 [b]	2.04	± 0.28 [b]
	H3K4me2	0.29	± 0.15 [b]	0.30	± 0.09 [b]	0.78	± 0.24 [a]	0.30	± 0.14 [b]
	H3K4me3	5.16	± 1.39 [ab]	4.83	± 0.95 [ab]	5.78	± 0.3 [a]	3.43	± 0.36 [b]
	Mock	0.53	± 0.16	0.27	± 0.09	0.03	± 0.03	0.12	± 0.1
VRN1	H3	17.74	± 1.04 [a]	15.97	± 1.81 [ab]	14.73	± 0.3 [b]	9.99	± 0.36 [c]
	H3K27me3	17.33	± 0.34 [a]	15.11	± 1.49 [ab]	15.71	± 1.24 [ab]	10.48	± 0.62 [b]
	H3K4me2	2.16	± 0.6 [ab]	2.12	± 0.16 [ab]	3.63	± 0.23 [a]	1.84	± 1.11 [b]
	H3K4me3	42.85	± 4.84 [b]	29.06	± 4.21 [c]	88.81	± 5.86 [a]	44.93	± 3.28 [b]
	Mock	0.54	± 0.13	0.28	± 0.12	0.15	± 0.05	0.16	± 0.01

Table S3: Gene ontology analysis of chilling-responsive genes.

	GO term	Description	Input	BG/Ref	p-value	FDR
**Shared, different expression**	GO:0001071	F nucleic acid binding transcription factor activity	5	478	0.0022	0.028
	GO:0003700	F transcription factor activity, sequence-specific DNA binding	5	478	0.0022	0.028
**Unique to naive**	GO:0001071	F nucleic acid binding transcription factor activity	32	478	2.50E-05	0.0053
	GO:0003700	F transcription factor activity, sequence-specific DNA binding	32	478	2.50E-05	0.0053
**Unique to primed**	GO:0044710	P single-organism metabolic process	48	1966	1.50E-07	5.20E-05
	GO:0044699	P single-organism process	62	2940	2.20E-07	5.20E-05
	GO:0043436	P oxoacid metabolic process	13	322	0.0001	0.014
	GO:0055114	P oxidation-reduction process	28	1159	0.00012	0.014
	GO:0006082	P organic acid metabolic process	13	358	0.00027	0.026
	GO:0044763	P single-organism cellular process	32	1544	0.00058	0.046
	GO:0003824	F catalytic activity	97	6501	0.00018	0.026
	GO:0016491	F oxidoreductase activity	30	1274	0.00011	0.026
**Shared, same expression**	GO:0031326	P regulation of cellular biosynthetic process	38	1085	6.10E-07	2.30E-05
	GO:0050789	P regulation of biological process	47	1470	2.90E-07	2.30E-05
	GO:2000112	P regulation of cellular macromolecule biosynthetic process	38	1085	6.10E-07	2.30E-05
	GO:0019219	P regulation of nucleobase-containing metabolic process	38	1072	4.60E-07	2.30E-05
	GO:0009889	P regulation of biosynthetic process	38	1085	6.10E-07	2.30E-05
	GO:0050794	P regulation of cellular process	47	1437	1.50E-07	2.30E-05
	GO:1903506	P regulation of nucleic acid-templated transcription	38	1058	3.30E-07	2.30E-05
	GO:2001141	P regulation of RNA biosynthetic process	38	1058	3.30E-07	2.30E-05
	GO:0051252	P regulation of RNA metabolic process	38	1059	3.40E-07	2.30E-05
	GO:0006355	P regulation of transcription, DNA-templated	38	1058	3.30E-07	2.30E-05
	GO:0010556	P regulation of macromolecule biosynthetic process	38	1085	6.10E-07	2.30E-05
	GO:0051171	P regulation of nitrogen compound metabolic process	38	1092	7.10E-07	2.40E-05
	GO:0065007	P biological regulation	47	1527	8.60E-07	2.50E-05
	GO:0010468	P regulation of gene expression	38	1101	8.70E-07	2.50E-05
	GO:0031323	P regulation of cellular metabolic process	38	1110	1.00E-06	2.70E-05
	GO:0080090	P regulation of primary metabolic process	38	1110	1.00E-06	2.70E-05
	GO:0060255	P regulation of macromolecule metabolic process	38	1130	1.60E-06	3.80E-05
	GO:0019222	P regulation of metabolic process	38	1136	1.80E-06	4.10E-05
	GO:0097659	P nucleic acid-templated transcription	39	1195	2.30E-06	4.70E-05
	GO:0006351	P transcription, DNA-templated	39	1195	2.30E-06	4.70E-05
	GO:0032774	P RNA biosynthetic process	39	1198	2.50E-06	4.80E-05
	GO:0034654	P nucleobase-containing compound biosynthetic process	40	1272	4.10E-06	7.60E-05
	GO:0018130	P heterocycle biosynthetic process	40	1336	1.30E-05	0.00022
	GO:0019438	P aromatic compound biosynthetic process	40	1335	1.20E-05	0.00022
	GO:1901362	P organic cyclic compound biosynthetic process	40	1387	2.90E-05	0.00048
	GO:0016070	P RNA metabolic process	40	1495	0.00015	0.0023
	GO:0043170	P macromolecule metabolic process	87	4365	0.00052	0.0078
	GO:0044238	P primary metabolic process	101	5315	0.00076	0.011
	GO:0071704	P organic substance metabolic process	104	5571	0.0012	0.016
	GO:0044260	P cellular macromolecule metabolic process	77	3862	0.0013	0.018
	GO:0044271	P cellular nitrogen compound biosynthetic process	42	1799	0.0016	0.021
	GO:0090304	P nucleic acid metabolic process	40	1708	0.0019	0.025

	GO:0008152	P metabolic process	127	7223	0.0022	0.028
	GO:0007154	P cell communication	11	280	0.0033	0.04
	GO:0001071	F nucleic acid binding transcription factor activity	26	478	1.70E-08	2.30E-06
	GO:0003700	F transcription factor activity, sequence-specific DNA binding	26	478	1.70E-08	2.30E-06
	GO:0043167	F ion binding	45	1701	6.70E-05	0.0059
	GO:0043169	F cation binding	42	1601	0.00015	0.0079
	GO:0046872	F metal ion binding	42	1594	0.00014	0.0079
	GO:0005509	F calcium ion binding	7	98	0.0008	0.035

Table S4: Chilling-responsive genes common to naïve and primed responses that show transcriptional memory (TM) and their categorization as diurnal-freezing responsive genes.

	Hypersensitive TM	Hyposensitive TM	**Total**	in diurnal-freezing
complex-convergent (U2A)	2	14	**18**	memory (59%)
transient (T3)	1	6	**7**	
complex-divergent (U2B, D2A)	1	1	**2**	
stable (U1, D1)	8	9	**17**	no memory (41%)
late-response (L1)	1	0	**1**	
NA / chilling-specific	4	2	**6**	
**Total**	**17**	**33**	**50**	
	34%	66%		
